# Transcriptome-wide Identification of Nine Tandem Repeat Protein Families in Roselle (*Hibiscus sabdariffa* L.)

**DOI:** 10.21315/tlsr2024.35.3.6

**Published:** 2024-10-07

**Authors:** Christina Seok Yien Yong, Nur Atheeqah-Hamzah

**Affiliations:** Department of Biology, Faculty of Science, Universiti Putra Malaysia, Jalan UPM, 43400 Serdang, Selangor, Malaysia

**Keywords:** Abiotic Stress, Biotic Stress, Protein-Protein Interaction, Roselle Calyx, Tandem Peptide Repeat, Tekanan Abiotik, Tekanan Biotik, Interaksi Protein-Protein, Kelopak Roselle, Ulangan Peptida Tandem

## Abstract

Plants are rich in tandem repeats-containing proteins. It is postulated that the occurrence of tandem repeat gene families facilitates the adaptation and survival of plants in adverse environmental conditions. This study intended to identify the tandem repeats in the transcriptome of a high potential tropical horticultural plant, roselle (*Hibiscus sabdariffa* L.). A total of 92,974 annotated *de novo* assembled transcripts were analysed using *in silico* approach, and 6,541 transcripts that encoded proteins containing tandem repeats with length of 20–60 amino acid residues were identified. Domain analysis revealed a total of nine tandem repeat protein families in the transcriptome of roselle, which are the Ankyrin repeats (ANK), Armadillo repeats (ARM), elongation factor-hand domain repeats (EF-hand), Huntingtin, elongation factor 3, protein phosphatase 2A, yeast kinase TOR1 repeats (HEAT), Kelch repeats (Kelch), leucine rich repeats (LRR), pentatricopeptide repeats (PPR), tetratricopeptide repeats (TPR) and WD40 repeats (WD40). Functional annotation analysis further matched 6,236 transcripts to 1,045 known proteins that contained tandem repeats including proteins implicated in plant development, protein-protein interaction, immunity and abiotic stress responses. The findings provide new insights into the occurrence of tandem repeats in the transcriptome and lay the foundation to elucidate the functional associations between tandem peptide repeats (TRs) and proteins in roselle and facilitate the identification of novel biotic and abiotic response related tandem repeats genes that may be useful in breeding improved varieties.

## Abstract (fr)

**Abstrak:**

Tumbuhan adalah kaya dengan protein yang mengandungi ulangan peptida tandem. Ia dipostulasikan bahawa kejadian famili gen ulangan tandem ini membantu adaptasi dan kemandirian tumbuhan dalam keadaan persekitaran mudarat. Kajian ini ingin mengenal pasti ulangan tandem di dalam transkriptom satu tumbuhan hortikultur tropikal berpotensi tinggi, roselle (*Hibiscus sabdariffa* L.). Sejumlah 92,974 transkrip beranotasi berhimpun secara *de novo* dianalisis menggunakan kaedah *in silico*, dan 6,541 transkrip yang mengkodkan protein yang mengandungi ulangan tandem dengan kepanjangan 20–60 residu asid amino dikenal pasti. Analisis domain mendedahkan sejumlah sembilan famili protein ulangan tandem di dalam roselle, iaitu *Ankyrin repeats* (ANK), *Armadillo repeats* (ARM), *elongation factor-hand domain repeats* (EF-hand), *Huntingtin, elongation factor 3, protein phosphatase 2A, yeast kinase TOR1 repeats* (HEAT), *Kelch repeats* (Kelch), *leucine rich repeats* (LRR), *pentatricopeptide repeats* (PPR), *tetratricopeptide repeats* (TPR) dan WD40 *repeats* (WD40). Analisis penganotasian fungsian seterusnya memadankan 6,236 transkrip kepada 1,045 protein diketahui yang mengandungi ulangan tandem termasuk protein yang terlibat dalam perkembangan, interaksi antara protein, imuniti semulajadi, dan tindak balas tekanan abiotik tumbuhan. Penemuan ini memberikan pandangan baharu terhadap kejadian ulangan tandem di dalam transkriptom roselle dan menyediakan asas untuk menjelaskan asosiasi fungsian antara ulangan peptida tandem dan protein di dalam roselle, dan membantu pengenalpastian gen ulangan tandem novel yang mungkin berguna dalam pembiakan varieti yang lebih baik.

**Highlights**
- Nine tandem repeat protein families were identified in the calyx transcriptome of roselle, namely the Ankyrin repeats (ANK), Armadillo repeats (ARM), elongation factor-hand domain repeats (EF-hand),Huntingtin, elongation factor 3, protein phosphatase 2A, yeast kinase TOR1 repeats (HEAT), Kelch repeats (Kelch), leucine rich repeats (LRR),pentatricopeptide repeats (PPR), tetratricopeptide repeats (TPR) and WD40 repeats (WD40).
- Transcriptome dataset could be a valuable source to discover tandem repeat protein families in the functional fraction of genome. A total of 1,045 tandem repeat-containing proteins implicated in various biological processes including plant development, protein-protein interaction, innate immunity, and abiotic stress responses were discovered.
- Biotic and abiotic response related tandem repeats genes for potential application in breeding improved varieties.

## INTRODUCTION

*Hibiscus sabdariffa* L., commonly known as roselle, is a multi-purpose tropical and subtropical shrub from the family of Malvaceae that possesses great therapeutic potentials. It is a valuable medicinal crop that can be cultivated in arid and semi-arid regions due to its high drought resilience. It has been used traditionally as folk medicines for decades particularly in regions where modern medicines are not readily accessible (Riaz & Copra 2018). Nonetheless, many scientific researches have reported the numerous beneficial properties of the extracts from roselle, including antibacterial, nephro-protective, hepato-protective, anti-diabetic and anti-hypertensive among others in recent years (Da-Costa-Rocha *et al*. 2014; von Dentz *et al*. 2021). Thus, it holds nutraceutical and pharmaceutical significance for industrial uses (Jamini & Aminul-Islam 2021), and is also frequently used as a functional ingredient in the food and beverages industries (Cid-Ortega & Guerrero-Beltrán 2015). The global roselle market size is expected to reach USD252.6 million by 2030 with a *compound annual growth rate* of 7.5% from 2021 to 2030. The calyx is the most valued part of the plant attributable to its rich content of phytochemicals such as phenolic compounds, anthocyanins and organic acids (Morales-Luna *et al*. 2019). While the phytochemicals and their bioactivities have warranted roselle considerable amount of attention from the industry players and scientific communities, the genetic aspects of this plant remain mostly unexplored.

Tandem peptide repeats (TRs) in proteins are generally made of conserved block of amino acids and found abundantly in the proteomes of different organisms across all domains of life. The tandem repeat gene families are found copiously in the genomes of eukaryotes in comparison to prokaryotes (Delucchi *et al*. 2020). Plants house the most TRs, and their occurrences are anticipated to facilitate the adaptation and survival of plants in unfavourable environmental conditions. The massive expansion of TR gene families in land plants over the course of evolution has supported possible roles in plant growth, development, and survival (Sharma & Pandey 2016). Thus, studying the TRs and their association with functional proteins is essential to understand plant’s adaptability. These tandem repeat regions provide a rich source for genetic variability with a wide range of possible genotypes at a given locus, which confers variability in adaptive ability in plants (Nithianantharajah & Hannan 2007). Although a substantial number of proteins that contains these adjacently repeated amino acid sequences have been identified, the biological significance for this association is still not entirely understood in plants.

Tandem repeats in protein-coding genes are mainly derived from internal gene duplication events (Luo & Nijveen 2014). These tandem repeats can be described based on the length of the repeating motif, the number of repeated units and/or the similarity among the repeat units (Delucchi *et al*. 2020). Tandem repeats with motif length of 20–60 amino acid residues are of particular interest to researchers because these TRs can function as structurally independent units and are likely to be folded as part of a protein domain displaying complex patterns as functional domain repeats (Jernigan & Bordenstein 2015). Examples of TRs in this category including leucine rich repeats (LRR, 20–30 amino acids), pentatricopeptide repeats (PPR, ~35 amino acid) and Armadillo repeats (ARM) (~40 amino acid). Studies have demonstrated that TR-containing proteins play many important biological roles in diverse organisms. For instances, LRR-containing proteins are associated with innate immunity in plants and animals (Ng & Xavier 2011), tetratricopeptide repeats (TPR) in proteins are known to mediate protein-protein interactions and formation of protein complexes, while PPR-containing proteins involve in organelle biogenesis and function, are fundamentally important for biological processes such as photosynthesis and plant development (Barkan & Small 2014). Although progresses have been achieved in some important plants such as *Arabidopsis thaliana* and *Oryza sativa* (Sharma & Pandey 2016), TRs remain largely understudied in most plant species.

To the authors’ best knowledge, no work has reported the TRs in roselle including their occurrence and association with functional proteins. This study had analysed an in-house calyx transcriptome dataset of roselle and identified the transcripts that encode proteins containing TRs with length of 20–60 amino acid residues. All the TR gene families identified and their associated proteins are described. Subsequently, the three most abundant TR gene families in roselle are discussed and the crucial roles of TR gene families in plant’s responses to biotic and abiotic stresses are deliberated. These findings enhance the understanding of TRs in roselle, their potential functions, and facilitate the identification of novel biotic and abiotic response related TR gene families for their use in breeding improved plant varieties.

## MATERIALS AND METHODS

The preparation of plant materials, RNA extraction, RNA-sequencing library, next generation sequencing (NGS) and preliminary data analysis were performed in a previous study (Hamzah *et al*. 2022), and are briefly described in the next subsections.

### Samples Preparation, RNA Extraction, RNA-Sequencing Library Preparation and Sequencing

*Hibiscus sabdariffa* var UMKL (a red variety registered as ‘HS2’ in the National Crop List of Malaysia) was used in this study. The planting material was obtained from the Department of Agriculture Terengganu, Malaysia. Mature calyxes were collected from six healthy individual roselle plants. The calyces were collected at maturation Stages III and IV according to number of days post-anthesis (DPA) as described in the guidelines provided by the Federal Agricultural Marketing Authority (FAMA) under the Ministry of Agriculture and Food Industry, Malaysia. These two maturation stages are particularly important in roselle cultivation because these stages are often associated with the quality of the calyces, and determine calyx harvesting time (Hamzah *et al*. 2022). Three biological replicates each were harvested on 32nd DPA (Stage III) and 59th DPA (Stage IV). The calyx samples were submerged in liquid nitrogen immediately upon collection and stored at −80°C until use. RNA samples were extracted from the calyx tissues using a *RNeasy Plant Mini Kit* (Qiagen, Germany) according to the manufacturer’s instructions. The quantity and quality of the RNA extracts were subsequently determined using Qubit Fluorometer 2.0 (Life Technologies Corporation, Carlsbad, US), Agilent 2100 Bioanalyzer (Agilent Technologies, Germany) via a Pico Chip and 1% agarose gel electrophoresis. Six sequencing libraries were prepared using ScriptSeq™ v2 RNA-Seq Library preparation kit (Epicentre, Madison, US) according to the manufacturer’s protocols. The sequencing was performed on an Illumina NextSeq 500 platform to generate 76 bp paired-end reads using the service provided by ScienceVision Sdn. Bhd., Selangor, Malaysia.

### Preliminary Data Analysis of Raw RNA Sequencing Reads

Raw reads were screened against the PhiX genome sequence to remove sequencing reads belonging to the sequencing control library using Bowtie2 version 2.2.3 (Langmead & Salzberg 2012). The sequencing reads were then subjected to adapters removal and base quality trimming (Q > 30). Bases with low quality scores and trimmed reads of less than 35 bp were discarded with its pair. Adapter removal, base quality trimming and discard of short reads were performed using BBDuk (BBTools version 36, http://jgi.doe.gov/data-and-tools/bbtools/). The sequencing short reads were submitted to National Center for Biotechnology Information Short Read Archive (NCBI-SRA) under the BioProject number PRJNA664826 with the accession numbers: SRX9171164, SRX9171165, SRX9171166 (Stage III), and SRX9171161, SRX9171162, SRX9171163 (Stage IV).

### Assembly, Functional Annotation, and Identification of TRs

The clean reads were assembled (Hamzah *et al*. 2022) and used in the current study for TRs identification. Good quality reads were *de novo* assembled into transcripts representing full or partial fragment of the transcriptome using Trinity Version 2.2.0 (Haas *et al*. 2013). The assembled transcripts were searched for protein coding region using TransDecoder version 2.0.1 (https://transdecoder.github.io). Subsequently, the transcript sequences and the predicted peptide sequences were subjected to sequence similarity search using BLAST version 2.2.31+ (Camacho *et al*. 2009) with an *E*-value cut-off of 10^−6^ to protein sequences of known functions available in the SwissProt database (Bairoch & Apweiler 2000), and scanned against protein domain motifs in the Protein Family database (Finn *et al*. 2014) using HMMER version 3.1b2 (Finn *et al*. 2011) with a GA bit scores and *E*-value cut-off of 10^−5^, respectively. Tandem repeat families containing tandem repeat with lengths of 20–60 amino acid residues were identified.

## RESULTS

### Good Quality Sequencing Reads and Transcript Assembly Statistics

The sequencing of the six calyx tissue RNA libraries generated a total of 237,321,698 good quality reads (Table 1), after data quality assessment (Hamzah *et al*. 2022). *De novo* assembly of these good quality reads yielded 221,334 contigs with minimum contig length of 161 bp, maximum contig length of 25,718 bp, and N50 score of 491 bp (Table 2). Each of the samples have between 89%–99% reads mapped to the *de novo* assembled transcriptome, signifying that most of the sequencing reads have been assembled and accounted for.

### Tandem Repeat Gene Families Identified

**Figure 1:**
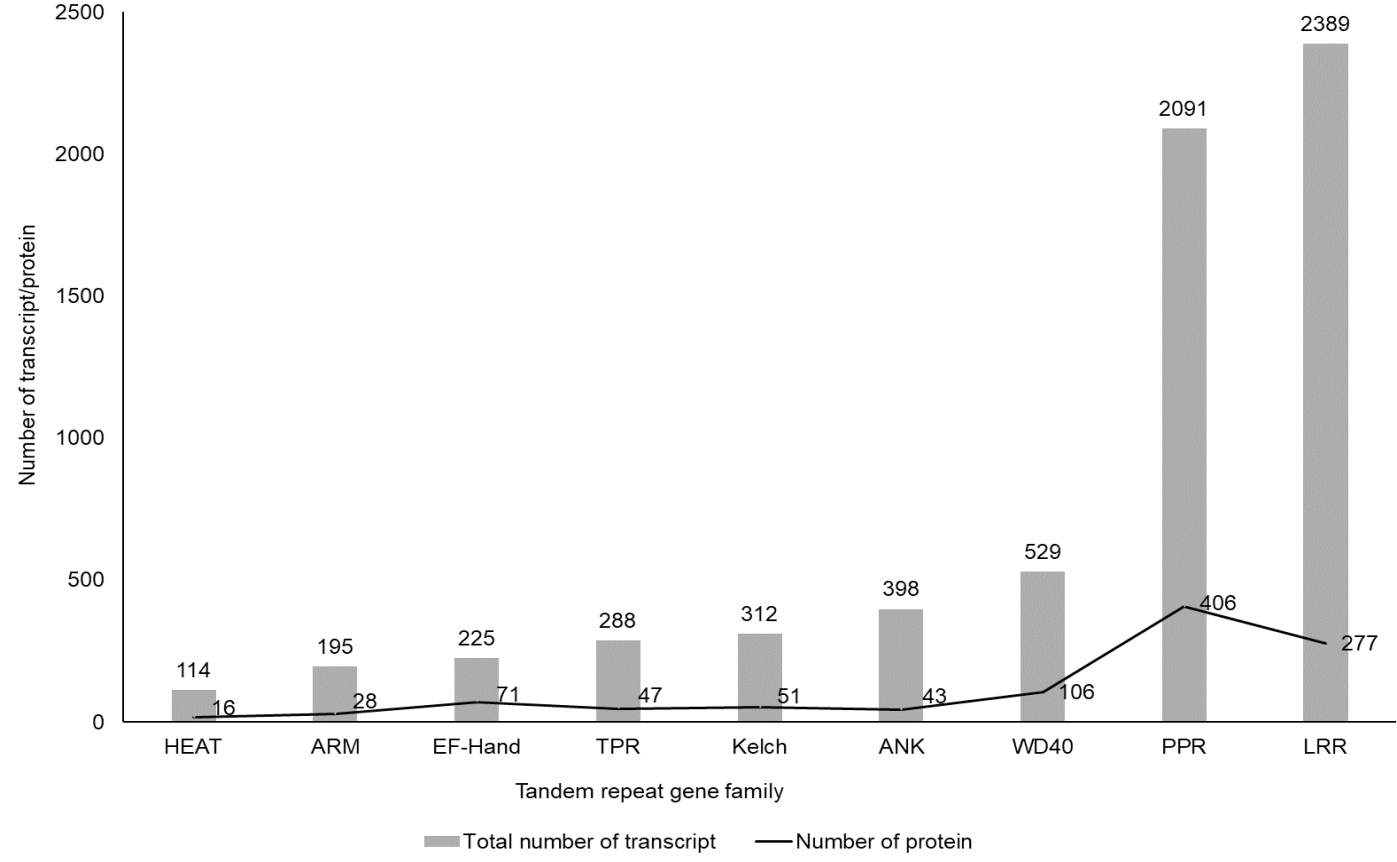
Tandem repeat gene families, the number of TR-containing proteins and the number of associated transcripts identified in the calyx transcriptome of roselle.

In the absence of a genome sequence, the calyx transcriptome generated from the authors’ previous study (Hamzah *et al*. 2022) was utilised to identify novel TR gene families present in roselle. Although the TR gene families identified here do not represent the complete collection of these gene families in roselle, it provides a glimpse of the TR gene families present in the functional genome fraction of this plant for the first time. This study analysed a total of 92,974 annotated *de novo* assembled transcripts and identified 6,541 transcripts that encode protein sequences containing tandem repeats (TRs) with the lengths of 20–60 amino acid residues. These transcripts corresponded to nearly 7.1% of the total annotated transcripts analysed. Nine TR gene families were identified, namely LRR, PPR, WD40 repeats (WD40), Ankyrin repeats (ANK), TPR, Kelch repeats (Kelch), elongation factor-hand domain repeats (EF-hand), ARM, and Huntingtin, elongation factor 3, protein phosphatase 2A, yeast kinase TOR1 repeats (HEAT). LRR and PPR gene families, represented by 2,389 and 2,091 transcripts, respectively, were the two more abundant TR families found in this study. In contrast, only 114 transcripts were related to proteins containing HEAT, making this TR the least common repeat gene family found in the roselle’s transcriptome. Functional annotation further linked 6,236 of the transcripts to 1,045 TR-containing proteins (Fig. 1). The remaining 305 transcripts also encoded amino acid sequences containing TRs, but they were either matched to uncharacterised proteins or did not match to any known proteins searched. The TRs identified, their associated proteins and the number of transcripts associated with each of the protein are displayed in Supplementary Tables S1 to S9. The TR-containing proteins identified in this study signified possible correlation between these TRs with specific functional proteins.

### Tandem Repeat Gene Families and Associated Proteins

#### Leucine rich repeats (LRR)

LRR is the most abundant tandem repeats identified from the roselle’s calyx transcriptome. A total of 277 known proteins that encompassed LRR were identified from 2,389 transcripts. Out of that, 108 proteins were identified as LRR receptor-like protein kinases (LRR-RLK). Among the LRR-RLK, LRR receptor-like serine/threonine protein kinases with 69 different proteins discovered (Refer Supplementary Table S1 for details) were the major type of LRR-RLK. Examples of LRR-RLK serine/threonine protein kinases identified were LRR receptor-like serine/threonine BAM1, LRR receptor-like serine/threonine EFR, LRR receptor-like serine/threonine ERECTA, LRR receptor-like serine/threonine RPK2, and LRR receptor-like serine/threonine FEI1. In addition, 39 non-serine/threonine types of LRR-RLK were also found. For instances, the LRR receptor-like protein kinases CORYNE, LRR receptor-like protein kinases TDR, LRR receptor-like protein kinases PXL2, LRR receptor-like protein kinases HAIKU2, and LRR receptor-like protein kinases HSL1. Furthermore, LRR receptor protein kinase (LRR-RK) with 23 proteins found contributed almost 21% to the overall LRR-kinases identified. Some of the LRR-RK proteins identified included protein brassinosteroid insensitive 1, brassinosteroid LRR receptor kinase, LRR receptor protein kinase EMS1, LRR receptor protein kinase CLAVATA1, serine/threonine protein kinase BRI1-like, and somatic embryogenesis receptor kinase 1.

In addition to LRR-RLK and LRR-RK, a considerable number of disease resistance/susceptibility proteins (55 proteins) were also found to comprise LRR, which corresponded to approximately 19% of the total LRR-containing proteins identified. The disease resistance RPP13-like proteins, disease resistance RPP8-like proteins, disease resistance protein RPS, and disease resistance protein RGA were among the more common ones found. The identification of these LRR-containing disease related proteins insinuated possible functional association between the LRR and these disease related proteins. Apart from that, many LRR-containing F-box proteins (54 proteins) were discovered in this study, such as the EIN3-binding F-box proteins, F-box protein FBW2, and F-box protein SKIP. Other LRR-containing proteins also included the plant intracellular RAS-related LRR proteins, LRR extensin-like proteins, and transport inhibitor response proteins.

#### Pentatricopeptide repeats (PPR)

PPR, the second most abundant TR family found in the calyx transcriptome, was represented by 2,091 transcripts. Functional annotation assigned 96.3% of the transcripts to 395 PPR-containing protein homologs of *A. thaliana* such as the PPR-containing proteins At1g62930, At1g71210, At1g73710 and At1g0990 (Refer Supplementary Table S2 for details). However, the specific functions of most of these proteins have not been corroborated. Thus, not much inference can be made pertaining to their functions. On the other hand, only 60 transcripts (less than 3% of the 2,091 transcripts) were annotated to 11 specific proteins with known functions, which were the PPR-containing protein MRL1, PPR protein for germination on NaCl, protein nuclear fusion defective 5, reticulon-like protein B22, PPR-containing protein DOT4, PPR-containing protein ELI1, two transcription factor proteins, proteinaceous RNase P1, PPR-containing protein PNM, and PPR-containing protein OTP51. Meanwhile, 16 transcripts showed no match to any proteins in the databases searched in this study. In total, 406 PPR-containing proteins (including those that matched to specific proteins and protein homologs of *Arabidopsis*) were discovered.

#### WD40 repeats (WD40)

A total of 529 transcripts were found to encode amino acid sequences comprising WD40. Functional annotation linked 452 transcripts (~85%) to 106 known proteins containing the WD40. However, the remaining 77 transcripts were either matched to uncharacterised proteins or showed no match to any proteins in the database searched. The Katanin p80 WD40-containing subunit B1 homolog and TOPLESS-related proteins represented by 38 and 39 transcripts, respectively, were the two more abundant WD40-containing proteins identified in this study (Refer Supplementary Table S3 for details). Other WD40-containing proteins identified were the WD-40-containing proteins MSI and autophagy-related proteins. Transcriptional related proteins such as the transcription initiation factor TFIID, transcriptional corepressor LEUNIG, zinc finger CCCH domain-containing proteins, RNA processing proteins such as the pre-mRNA-processing factors, and ribosome biogenesis proteins such as U3 small nucleolar RNA-associated proteins and U4/U6 small nuclear ribonucleoprotein PRP4-like protein were also found to contain WD40. In addition, several WD40-containing proteins associated with cell cycle were also discovered.

#### Ankyrin repeats (ANK)

Forty-three ANK-containing proteins were identified from 398 annotated transcripts. Most of the transcripts were annotated with known functional proteins except for four transcripts that did not match to any known proteins. The ADP-ribosylation factor GTPase-activating proteins consisting of AGD1–AGD9 (96 transcripts) formed the biggest group of ANK-containing proteins found in this study (Refer Supplementary Table S4 for details). The E3 ubiquitin-protein ligases represented by 80 transcripts were also among the more abundant group of ANK-containing proteins identified. In addition, several regulatory proteins that encompassed the ANK were found, such as regulatory protein NPR 1, 2, 4 and 5. Other ANK-containing proteins identified were protein S-acyltransferase 24, ANK protein SKIP35, BTB/POZ domain-containing protein At2g04740 and ANK domain-containing protein EMB506.

#### Kelch repeats (Kelch)

A total of 312 transcripts were found to encode amino acid sequences containing Kelch. Out of that, 301 transcripts matched to either known proteins (24 matches) or protein homologs from *A. thaliana* (27 matches), while 11 transcripts did not match to any proteins in the database (Refer Supplementary Table S5 for details). F-box/Kelch proteins (32 proteins represented by 195 transcripts) were the main type of proteins found. Among them, the F-box/Kelch SKIP proteins (SKIP4, SKIP6, SKIP11, SKIP25 and SKIP30) were the most abundant group of Kelch-containing proteins identified. The remaining 106 transcripts were annotated to various Kelch-containing proteins such as Acyl-CoA-binding domain-containing protein 4 and 5, Adagio proteins 1 and 3, serine/threonine-protein phosphatase BSL1, 2, and 3, RING finger protein B, and F-box protein AFR.

#### Tetratricopeptide repeats (TPR)

This study identified 47 known proteins containing TPR from 288 annotated transcripts. The peptidyl-prolyl cis-trans isomerases consisted of six different isomerases (represented by 27 transcripts) were the most abundant type of TPR-proteins found (Refer Supplementary Table S6 for details). Three Hsp70–Hsp90 organising proteins (represented by 24 transcripts), three anaphase-promoting complex subunits (represented by 8 transcripts), and two UDP-N-acetylglucosamine--peptide N-acetylglucosaminyltransferases (represented by 25 transcripts) were also found. This study also identified several envelope or membrane associated TPR-proteins such as the outer envelope proteins, alpha-soluble N-ethylmaleimide-sensitive factor (NSF) attachment protein and Translocon complex. Other TPR-containing proteins included the peroxisome biogenesis protein 5, protein SGT1 homolog, ethylene-overproduction protein 1, clustered mitochondria protein, and protein CTR9 homolog. Fifty-six transcripts out of the 288 transcripts were either matched to uncharacterised proteins or did not match to any known proteins in the searched databases in this study.

#### Elongation factor-hand domain repeats (EF-hand)

Seventy EF-hand-containing proteins were identified from 225 transcripts. Majority of these EF-hand-containing proteins were calcium-dependent protein kinases (CDPKs 17 proteins represented by 46 transcripts) and calcium-binding proteins (15 proteins represented by 29 transcripts). Apart from that, five calcineurin B-like (CBL) proteins, three calmodulin (CaM) and seven calmodulin-like (CML) proteins were also found. Other less abundant EF-hand-containing proteins found were the calcineurin subunit B, caltractin, calumenin, serine/threonine protein phosphatase 2A regulatory subunits, external alternative NAD(P)H-ubiquinone oxidoreductase B and two pore potassium channel proteins (Refer Supplementary Material Table S7 for details). Nearly 73% of the EF-hand containing proteins identified in this study were either calcium-associated or calcium-dependent proteins signifying their potential involvement in the regulation of calcium signalling.

#### Armadillo repeats (ARM)

A total of 28 proteins containing ARM were predicted from 195 transcripts. The U-box-domain proteins (13 proteins) represented by 55 transcripts were the most abundant group of ARM-containing proteins found in this study. This was followed by the ARM -containing kinesin-like proteins (three proteins represented by 41 transcripts) and importin subunit alpha proteins (six proteins represented by 39 transcripts). The remaining six ARM-containing proteins were proteins ARABIDILLO 1 and 2, ARM protein interacting with ABF2, ARM-containing protein LFR, vacuolar protein 8 and phospholipase A I (Supplementary Table S8). Meanwhile, 14 transcripts which also encode amino acid sequences containing ARM repeats, they did not match to any known proteins in this study.

#### Huntingtin, elongation factor 3, protein phosphatase 2A, yeast kinase TOR1 repeats (HEAT)

HEAT was the rarest TRs found in the calyx transcriptome of roselle, with only 114 transcripts annotated to HEAT-containing proteins. Compared with other TRs, the number of HEAT-containing proteins (16 proteins) found in this study were relatively lower. Among them, protein SHOOT GRAVITROPISM 6 has the highest number of transcripts (52 transcripts) representing it (Supplementary Table S9). In addition, several regulatory or transcription related HEAT-containing proteins were found such as the serine/threonine-protein phosphatase 2A 65 kDa regulatory subunit A beta isoform, 26S proteasome non-ATPase regulatory subunit initiation factor TFIID subunit 6. Two microtubule-associated proteins were also found to contain HEAT, namely the protein TORTIFOLIA1 and protein SPIRAL2-like. Other proteins such as the CLIP-associated protein, coatomer subunit gamma-2 protein, importin proteins and protein MOR1 that encompassed HEAT were also identified.

## DISCUSSION

Proteins encompass TRs are frequently associated with additional functional domains, which expands the functional diversity of proteins within the same gene family. Tandem repeats in proteins are frequently implicated in mediating protein-protein interactions. Thus, changes in these TR structural units will likely interfere with the protein functions (Parra *et al*. 2015). The occurrence of TRs was found to correlate positively with proteome size, which implies the probable association of TRs and protein’s functions (Schaper & Anisimova 2015). To date, about one-half of all TRs recognised are common protein domains. Despite that, the tandem repeat gene families remain undiscovered in most plants, and the occurrence or association of the TRs with protein functions are still inadequately studied. The reason could be due to the high sequence divergence in repeating units that often poses technical challenges in studying repeat gene families (Parra *et al*. 2015). It is further complicated by the lack of genome sequences for most non-model plant species including roselle. This study took advantage of the authors’ transcriptome dataset (Hamzah *et al*. 2022) to identify the TR-containing proteins in roselle. Although genome sequences give more comprehensive and accurate prediction of TRs occurrence, the current study has provided novel insights into the occurrence of TRs in the transcribed genome of roselle.

### Comparison of TR Family in Arabidopsis, Rice and Roselle

Whole genome analyses on *A. thaliana* and *O. sativa* had separately classified multigene repeat families in dicotyledon and monocotyledon, respectively. In *A. thaliana*, LRR was identified as the most abundant repeat proteins, followed by PPR and WD40 proteins. Meanwhile, ANK, TPR, ARM and Kelch proteins occurred at lower frequencies than the former three TR gene families and HEAT proteins were the least common in *Arabidopsis*. In *O*. *sativa*, PPR was the most common type of repeat proteins found, followed by LRR and WD40. As for ANK, TTR, ARM, Kelch and HEAT, similar levels of their relative abundances were observed in *O*. *sativa*, as in *A. thaliana* (Sharma & Pandey 2016). As for EF-hand, 250 and 243 EF-hand-containing proteins were reported in the whole genomes of *Arabidopsis* (Day *et al*. 2002) and *Oryza* (Boonburapong & Buaboocha 2007), respectively.

This present study also identified nine TRs gene families in the transcriptome of roselle, similar to those reported in the two model plants. Table 3 compares the number of TR-containing proteins identified from the genomes of *A.thaliana* and *O. sativa* reported in previous studies, and the transcriptome of roselle in this study. In comparison to the TR-containing proteins identified in the genomes of *Arabidopsis* and rice, the number of TR-containing proteins of the LRR-RLK, WD40, ANK, TPR, EF-hand and ARM repeat gene families found in this study were relatively lower. The number of Kelch-containing proteins discovered in this study fell between the counts observed in *Arabidopsis* and rice. While for the PPR and HEAT families, the numbers of proteins found were comparable with those reported in *Arabidopsis* and rice.

The relatively lower number of TR-containing proteins found in this study was not unexpected, given the extensive studies and availability of the complete genome sequences of the two model plant species. The disparity in the data size and data type used (i.e., genome vs transcriptome) likely contributed to the lower number of TR-containing proteins found in this study. In addition, the calyx tissue-specific nature of the data also possibly influenced and constrained the number of TR-containing proteins identified in this study. Despite data limitation, the calyx’s transcriptome has enabled the identification of nine TR gene families in roselle.

Although the number of TR-containing proteins found was lower in roselle compared to *Arabidopsis* and rice, it is worth noting the occurrence of tandem repeat proteins identified in roselle in terms of relative abundance of each TR family was generally comparable to that of the *Arabidopsis* and rice, except for EF-hand-containing proteins. Notably, LRR, PPR and WD40 were found to be the more abundant TRs families in all three species. The abundance of ANK, TPR, ARM, Kelch and HEAT proteins are lower than the former three, with HEAT proteins being the least common. However, there were only 70 EF-hand-containing proteins found in the transcriptome of roselle, in contrast to more than 200 EF-hand-containing proteins identified in the genomes of *Arabidopsis* and rice (Table 3). Apart from the differences in data sizes described above, the tissue- and maturation stage-specific nature of the transcriptome might have contributed to the discrepancy. Zeng *et al*. (2017) revealed that the number of EF-hand proteins in plant is directly influenced by tissue developmental stage and type. For instances, some EF-hand-containing *CML* proteins are expressed exclusively in the flower; while others expressed specifically in the root tissue. Though the TRs predicted from the transcriptome in this current study may not reflect the actual occurrence of TRs in the genome of roselle, the relative abundance of proteins in the TR families identified, from the most common to the rarest, were fundamentally comparable to those reported in the whole genome analysis of *A. thaliana* largely. This finding suggested that transcriptome data could be a valuable alternative to study TR gene family in non-model plants, providing insights to these important gene families in the absence of the genome sequences.

### The Three Most Abundant Tandem Repeat Families in Roselle

#### Leucine rich repeats (LRR)

LRR is the most prominent tandem repeat type discovered from the roselle’s calyx transcriptome. LRR is frequently associated with the mediation of protein-protein interaction of cell-cell communication and in plant innate immunity. Its slender, arc-shaped structure provides maximum surface area for protein-protein binding (Padmanabhan *et al*. 2009). Of the LRR-containing proteins identified in this study, more than half were LRR receptor-like protein kinases (LRR-RLK). The LRR-RLKs are the largest subgroup of the RLK family and represent a complex gene family in plants (Liu *et al*. 2017; Li *et al*. 2018). All LRR-RLK proteins possess the N-terminal LRR domain, a single transmembrane domain, and a C-terminal kinase domain, which are important for cellular signalling via interaction with various ligands (Dufayard *et al*. 2017; Chakraborty *et al*. 2019). In *Arabidopsis*, more than 600 RLKs were identified and out of those, 223 members are LRR-RLKs (Shiu *et al*. 2004; Wu *et al*. 2016). In rice, 1,100 RLKs had been identified with 292 members being the LRR-RLKs (Morillo & Tax 2006; Hwang *et al*. 2011). The expansion of the LRR-RLK gene family and their ubiquity in all land plants imply their importance in the evolution and survival of plants (Li *et al*. 2018). To date, only ~35% of the LRR-RLK members in *Arabidopsis* have been functionally assigned, and to a lesser extend in other species (Wu *et al*. 2016). The LRR receptor-like serine/threonine-protein kinases are the most common group of LRR-RLK found in this study. This group of LRR-RLKs interacts with a diverse group of proteins to affect a wide array of biological processes related to signalling in plants (Afzal *et al*. 2008). Examples of LRR receptor-like serine/threonine-protein kinases found in this study include LRR receptor-like serine/threonine RPK2 which is a regulatory factor for anther development (Mizuno *et al*. 2007), LRR receptor-like serine/threonine-protein kinase At3g14840 (also known as LYSM RLK1-interacting kinase 1) required for microbe associated molecular pattern-triggered innate immunity (Le *et al*. 2014), and LRR receptor-like serine/threonine ERECTA that regulates multiple development processes including flowering and stomatal cell differentiation (Shpak 2013; Pillitteri & Torii 2012).

This study also uncovered numerous transcripts that encode LRR-containing proteins that are related to disease resistance. Plant disease resistance genes are an essential component of plant’s genetic resistance mechanism. Majority of the immune receptors have the LRR domains, which display broad interaction surfaces and confer diverse classes of immune receptors with a platform to mediate protein-protein interactions (Padmanabhan *et al*. 2009; Ng & Xavier 2011; Jose *et al*. 2020). The highly mutable TRs enriched in LRR commonly found in R genes of plants are believed able to facilitate resistance to newly emerging pathogen (Schaper & Anisimova 2015). Furthermore, majority of disease resistance genes (R genes) in plants encode the nucleotide-binding site LRR proteins (NBS-LRR) that are actively involved in the detection of diverse plant pathogens (McHale 2006; Wan *et al*. 2012). NBS-LRR proteins are encoded by one of the most important gene families involved in plant immunity. Some of the NBS-LRR proteins discovered in this study were the disease resistance proteins RPP8, RPP13, RPS2, RPS4, RPS5 and RPM1. Based on past studies on *Arabidopsis*, RPP8 and RPP13 conferred resistance to downy mildew caused by *Peronospora parasitica* (Bittner-Eddy 2000; Mohr *et al*. 2010). RPS2, RPS4, RPS5 and RPM1 were the key NBS-LRR proteins that provide resistance against *Pseudomonas syringae* (Tao *et al*. 2000; Shao *et al*. 2016).

LRR-RLKs involved in the control of plant growth and development were also found in this study. For example, protein brassinosteroid-insensitive-1 regulates plant growth and development via its interaction with brassinosteroid, a common phytohormone in many plants (Friedrichsen *et al*. 2000; Navarro *et al*. 2015), LRR receptor protein kinase EMS1 and somatic embryogenesis receptor kinase 1 are involved in determining anther cell fate in plants (Li *et al*. 2017), whereas CLAVATA1 and CLAVATA 2 are implicated in flower development (Jones *et al*. 2021). LRR-containing F-box proteins are one of the largest regulatory protein superfamilies in plants involved in protein degradation and ubiquitination controlling crucial processes such as floral organogenesis, senescence, embryogenesis, hormonal responses and seedling development (Lechner *et al*. 2006). The LRR at the C-terminal domain of F-box proteins is important for target protein recognition and binding (Hong *et al*. 2021). In this study, 22 LRR-containing F-box proteins were identified in the transcriptome of roselle. For example, ethylene-insensitive binding F-box proteins (EIN3) is involved in ethylene signalling in plants by regulatingEIN3 or EIN3-like turnover (Binder *et al*. 2007). F-box protein FBW2 regulates theexpression of argonaute1 (AGO1) protein via proteolysis activities, and indirectly controls both micro RNA- and small interfering RNA-directed silencing in plants(Hacquard *et al*. 2022).

#### Pentatricopeptide repeats (PPR)

The abundance of PPR proteins found in this study agreed well with previous studies. PPR gene family is one of the largest and highly expanded nuclear-encoded protein families in terrestrial plants (Nakamura *et al*. 2012), with over 400 members in most sequenced plant species (Wang X *et al*. 2021). Many PPR proteins play prominent roles in plant growth and development (Qu *et al*. 2019). These proteins are fundamentally sequence-specific RNA-binding proteins that serve as important site recognition factors for organellar RNA processing events such as RNA cleavage, degradation, stability and splicing (Nakamura *et al*. 2012; Gutmann *et al*. 2020).

Breakthroughs in understanding PPR protein-RNA sequence recognition mechanisms have shed some lights on their involvement in regulating gene expressions of organellar transcripts (Barkan & Small 2014; Wang X *et al*. 2021). Thus, they have profound effects on organelle biogenesis and functions including photosynthesis, respiration and environmental responses (Barkan & Small 2014; Ren *et al*. 2020). This study identified PPR-containing proteins from the transcriptome of roselle that are involved in the transcriptional regulation of chloroplast and/or mitochondrial genes. For example, PPR-containing protein MRL1 acts on chloroplast gene and is essential for the production/stabilisation of the processed transcript (Johnson *et al*. 2010), PPR-containing protein ELI1 and PPR-containing protein DOT4 are required for RNA editing in chloroplast (Hayes *et al*. 2013), proteinaceous RNase P1 resides in mitochondria and chloroplast is necessary to support organellar function (Chen *et al*. 2019), PPR-containing protein OTP51 is needed for intron splicing in the chloroplast (de Longevialle *et al*. 2008), and PPR-containing protein PNM is involved in protein translation in mitochondria (Hammani *et al*. 2011). Mutational study on PPR protein-coding genes had also provided evidence that alteration to these genes caused dysfunction of mitochondria or/and chloroplasts, which resulted in growth retardation (Li *et al*. 2021).

#### WD40 repeats (WD40)

WD40 is an important tandem repeat family found in multitude of eukaryotic proteins. The WD40-containing proteins act as scaffolding molecules that manoeuvre activities of proteins involved in cellular, metabolic and molecular pathways (Mishra *et al*. 2012). In this study, these repeats were found to associate with various proteins in which many of them act as regulatory factors or proteins involved in cell division, chromatin organisation, and pre-mRNA processing. Among the WD40 proteins identified in this study, Katanin p80 WD40 repeat-containing subunit B1 homolog is the most abundant WD40-containing protein. Katanin is a heterodimer microtubule severing ATPase essential for the organisation and proper functions of the spindle, thus, regulates the progression of cell division and plane orientation (Luptovčiak *et al*. 2017). It is also critical for fundamental biological processes such as cell elongation and morphogenesis (Takáč *et al*. 2017). Other WD40-proteins involved in cell division included anaphase-promoting complex subunit essential in plant reproduction (Saleme *et al*. 2021) and mitotic checkpoint protein BUB3 required for gametophyte development (Lermontova *et al*. 2008).

TOPLESS proteins and TOPLESS-related proteins found in this study are among the most prominent transcriptional corepressors in plants (Martin-Arevalillo *et al*. 2017), which are involved in the regulations of hormone signalling (Dinesh *et al*. 2016) and interacts with WUSCHEL protein regulating plant stem cell homeostasis (Ikeda *et al*. 2009). The C-terminal WD40 on the TOPLESS proteins serve as the protein-protein interaction surface (Collins *et al*. 2019). Other transcriptional factors such as zinc finger CCCH domain-containing proteins are involved in mRNA processing (Peng *et al*. 2012), and transcriptional co-repressor LEUNIG and its homologs regulate cell fate specification and hormone signalling (Sitaraman *et al*. 2008). The WD40-containing proteins MSI, which were also relatively abundant in the transcriptome of roselle, are a group of histone binding WD40 proteins that form an essential part of a histone deacetylase complex that regulate gene expression (Mehdi *et al*. 2016). Several WD40 containing autophagy-related proteins (ATG16, ATG18a, ATG18c, ATG18d, ATG18f, ATG18g and ATG18h) were also found in this study. In general, ATGs are essential for maintaining cellular homeostasis and to prolong cell life under stress conditions. The evolutionary conserved ATG16 is one of the core autophagy proteins necessary for autophagosome formation and maturation (Xiong *et al*. 2019), while ATG18 was shown to respond to high salt and osmotic stress conditions (Wang H *et al*. 2021).

### Crucial Roles of TR Proteins Associated with Plant Resistance to Biotic and Abiotic Stresses for Breeding Resilient Plant Varieties

TR gene families are ubiquitously present in plant’s genome and play pivotal roles in a myriad of biological processes essential for plant growth, development, and responses to biotic and abiotic stresses. For examples, LRR-RLPs are involved in both plant development (Kruijt *et al*. 2005) and plant-pathogen interaction (Wulff *et al*. 2009). Similarly, ANK-containing proteins are instrumental for plant growth and development, and responses to a range of biotic and abiotic stresses (Yuan *et al*. 2013). Acyl-CoA-binding domain-containing protein 4, the most abundant Kelch-peptide repeat-containing protein found in this study, is involved in transporting oleoyl-CoA from the chloroplast to endoplasmic reticulum (Leung *et al*. 2004) and regulating drought tolerance (Lai & Chye 2021) in *Arabidopsis*. Consequently, genes that encode TR-containing proteins may be expressed to serve diverse purposes in plants during normal or/and unfavourable growing conditions. The ubiquity of TRs in plants is possibly caused by their positive selection during evolution attributable to their correlation with plant growth and development, and adaptation. It is hypothesised that the ability of TR proteins to establish interaction with multiple ligands in response to biotic and abiotic stress contributed to their selection during evolution (Sharma & Pandey 2016). Through *in silico* analysis, this study found that other than the HEAT families, all TR gene families discovered have some members implicated in plant’s responses to biotic and/or abiotic stresses. The identification of TR gene families and their respective proteins that are associated with plant’s response to biotic and abiotic stresses is essential to promote the application of these genes in breeding more resilient varieties (Gottin *et al*. 2021).136

For resistance to biotic stress, the identification of disease resistance LRR-containing proteins and LRR receptor-like serine/threonine protein kinase proteins strongly support the likely involvement of LRR domain in plant’s immunity proteins. The nucleotide binding-LRR proteins such as disease resistance protein TAO1 and disease resistance protein ADR2 found in this study are some of the important proteins responsible for pathogen detection and host defence (DeYoung & Innes 2006). Other than LRR proteins, TPR containing alpha-soluble NSF attachment protein also contributes to additive resistance in plant against pathogen (Lakhssassi *et al*. 2017), while TPR-protein SGT1 is required to induce host and non-host disease resistance in plants (Peart *et al*. 2002). The importin subunit alpha that was found in this study is a group of ARM-repeat proteins, they function as nuclear transport receptors that are implicated in nuclear translocation of immune regulatory proteins in animal and plant defence signalling (Wirthmueller *et al*. 2013). Phospholipase A 1 is also an ARM-containing protein, which confers resistance against biotic stress in plant (Canonne *et al*. 2011). ANK-containing proteins localised in the chloroplast are also involved in defence-related signalling in plants (Cao *et al*. 1997; Despres *et al*. 2001). Ankyrin-containing protein accelerated cell death 6 is a necessary defence response activator against virulent bacteria in plant (Lu *et al*. 2003); whereas regulatory proteins NPR1, 3 and 4 are the key receptors interacting with plant defence hormone salicylic acid, which play broad roles in plant immunity (Liu *et al*. 2020).

As for resistance against abiotic stress, this study identified several peptidyl-prolyl cis-trans isomerases and TPR repeat-containing thioredoxin TTL1 proteins, which were reportedly involved in osmotic stress responses in plants (Sharma *et al*. 2003; Rosado *et al*. 2006). This study also found other TPR proteins [Hsp70-Hsp90 organising proteins (HOP) 1, 2 and 3] that are particularly important for plant responses to heat stress. HOP functions as co-chaperones that mediate protein-protein interactions in plant responses to heat stress (Kurek *et al*. 1999; Toribio *et al*. 2020). The transcriptional response in *Arabidopsis* was tremendously altered in *hop1 hop2 hop3* triple mutant during acclimatisation to high temperature (Fernández-Bautista *et al*. 2018). Kelch-containing acyl-CoA-binding domain-containing protein 4 (ACBP4) is involved in regulating drought tolerance (Lai & Chye 2021) in *Arabidopsis*. ANK-containing protein At3g12360 had been found to confer salt-stress tolerance in *A. thaliana* (Sakamoto *et al*. 2008). Several main EF-hand calcium signal sensor proteins were found in this study, including CDPKs, CaM, CML and CBL proteins. These sensor proteins decipher the calcium signals triggered by environmental stimuli such as drought, heat, and salt stresses, and thus are important for plant abiotic stress responses (Pandey *et al*. 2015; Shi *et al*. 2018). LRR receptor-like kinase protein floral organ number1 (FON1), was demonstrated to increase drought tolerance in rice (Feng *et al*. 2014). Almost half of the ARM- containing proteins found in this study are U-box/ARM proteins such as U-box/ARM protein 18 and 19. These proteins play crucial roles in plant to achieve proteome plasticity in response to environmental stress (Mudgil *et al*. 2004).

There are also TR-proteins found in this study that respond to both abiotic and biotic stresses. Although LRR proteins are more often associated with plan’s immunity, many LRR-proteins are also involved in regulating plant’s responses to abiotic stress (Soltabayeva *et al*. 2022). Examples of LRR proteins that response to biotic and abiotic stresses found in this study are the LRR receptor-like kinases. Apart from LRR-proteins, other TR proteins such as the ANK-containing E3 ubiquitin-protein ligases, play essential roles in regulating specific protein ubiquitination associated with plant immunity (Gao *et al*. 2021) and abiotic stress responses (Shu & Yang 2017). Pentatricopeptide repeat protein for germination on NaCl (PGN) is also implicated in biotic and abiotic stress tolerance in plants (Laluk *et al*. 2011). The WD40-containing protein pleiotropic regulatory locus 1 is involved in regulating plant innate immunity and responses to environmental changes (Baruah *et al*. 2009).

The identification of TR-containing proteins responsible for plant’s resistance to abiotic and biotic stresses may elucidate the molecular mechanisms behind plant stress adaptations. Studies on the natural variability, evolution and functions of TR family genes are critical to identify novel stress-tolerance genes and provide the foundation for their application in breeding future climate-resilient varieties (Marone *et al*. 2013; Liao *et al*. 2017; Gottin *et al*. 2021). Furthermore, the discovery of highly mutable TRs enriched in LRRs that are commonly found in novel broad-spectrum R genes of plants and the NBS-LRR proteins, which provide effector triggered immunity in plants (Yu *et al*. 2022), could enhance the understanding of their function in disease resistance and further facilitate in producing disease-resistance varieties (Lee & Yeom 2015; Zhang *et al*. 2021).

## CONCLUSION

Tandem repeat often acts as a scaffold for substrate proteins, mediating protein-protein interactions that are fundamentally important from essential processes such as cell division to adaptive mechanisms such as responses to biotic and abiotic stresses in plants. This study identified nine TR families from the calyx tissue transcriptome of roselle and described their possible associated proteins. The study provides new insights to the occurrence and potential functions of the TR gene families in roselle, and thus established the fundamental knowledge for further studies to ascertain their biological functions in roselle. Given that the TR gene families prediction in this study was based on the calyx tissue transcriptome of roselle, the occurrence of the TRs might be influenced by the types of tissue studied and physiological state of the plant. Nonetheless, the relative abundance of TR gene families predicted corresponded well with those found in the genomes of *Arabidopsis* and rice, where LRR, PPR and WD40 are more abundant compared to other TR gene families. The TR-proteins associated with plant’s immunity and responses to abiotic stress found in this study, provide useful baseline data to explore novel candidate genes to facilitate breeding future tolerance varieties.

## Supplementary Information



## Figures and Tables

**Table 1:** Good quality reads generated from each of the roselle sample.

Sample	Good quality reads (pairs)
Calyx stage III (biological replicate 1)	38,507,642
Calyx stage III (biological replicate 2)	40,661,821
Calyx stage III (biological replicate 3)	41,267,234
Calyx stage IV (biological replicate 1)	40,981,601
Calyx stage IV (biological replicate 2)	35,605,342
Calyx stage IV (biological replicate 3)	40,298,058

Total	237,321,698

**Table 2:** Transcript assembly statistic of the roselle calyx’s transcriptome.

Parameter	Value
Total number of contigs	221,334
Minimum contig length (bp)	161
Minimum contig length (bp)	25,718
Average contig length (bp)	432
N50 score (bp)	491

Total contig length (bp)	95,824,738

**Table 3:** Comparison of TR-containing proteins predicted in the genomes of *Arabidopsis* and *Oryza*, and the number of TR-containing proteins identified in the transcriptome of roselle.

Tandem repeat	*A. thaliana*	*O. sativa*	*H. sabdariffa* (current study)
LRR-RLK	223 (Wu *et al*. 2016)	292 (Hwang *et al*. 2011)	108
PPR	458 (Colcombet *et al*. 2013)	491 (Chen *et al*. 2018)	406
WD40	237 (Van Nocker & Ludwig 2003)	200 (Ouyang *et al*. 2012)	106
Kelch	97 (Sun *et al*. 2007)	28 (Sun *et al*. 2007)	51
ANK	105 (Becerra *et al*. 2004)	175 (Huang *et al*. 2009)	43
TPR	177 (Wei & Han 2017)	216 (Wei & Han 2017)	47
EF-hand	250 (Day *et al*. 2002)	243 (Boonburapong & Buaboocha 2007)	70
ARM	108 (Mudgil *et al*. 2004)	158 (Sharma *et al*. 2014)	28
HEAT	17 (Sharma & Pandey 2016)	17 (Sharma & Pandey 2016)	16
