# Supplementary material for: Transcriptome-wide Identification of Nine Tandem Repeat Protein Families in Roselle (Hibiscus sabdariffa L.)
Source: Trop Life Sci Res. 2024 Oct 7;35(3):121–48. doi: 10.21315/tlsr2024.35.3.6 (PMC11507979; doi:10.21315/tlsr2024.35.3.6)
Supplement: Supplementary file 1 [file TLSR_35-3-121-suppl.pdf]

SUPPLEMENTARY MATERIALS

Transcriptome-wide Identification of Nine Tandem Repeat Protein Families in Roselle (*Hibiscus sabdariffa* L.)

Christina Seok Yien Yong* and Nur Atheeqah-Hamzah

Department of Biology, Faculty of Science, Universiti Putra Malaysia, Jalan UPM, 43400 Serdang, Selangor, Malaysia

*Corresponding author: chrisyong@upm.edu.my

Table S1: Leucine rich repeat-containing proteins identified in the calyx transcriptome of roselle.

No	Protein group	Protein	Number of transcripts
1.	LRR-RLK (serine/threonine)	LRR receptor-like serine/threonine-protein kinase At1g56130	64
2.		LRR receptor-like serine/threonine-protein kinase RPK2	45
3.		LRR receptor-like serine/threonine-protein kinase At1g07650	38
4.		Leucine-rich repeat receptor-like serine/threonine-protein kinase BAM1	35
5.		LRR receptor-like serine/threonine-protein kinase At1g63430	28
6.		LRR receptor-like serine/threonine-protein kinase At2g16250	24
7.		LRR receptor-like serine/threonine-protein kinase At1g53440	21
8.		LRR receptor-like serine/threonine-protein kinase At1g06840	24
9.		LRR receptor-like serine/threonine-protein kinase RFK1	22
10.		LRR receptor-like serine/threonine-protein kinase ERECTA	21
11.		LRR receptor-like serine/threonine-protein kinase EFR	20
12.		LRR receptor-like serine/threonine-protein kinase At4g08850	19
13.		LRR receptor-like serine/threonine-protein kinase At1g74360	19
14.		LRR receptor-like serine/threonine-protein kinase BIR2	16
15.		LRR receptor-like serine/threonine-protein kinase GSO1	16
16.		LRR receptor-like serine/threonine-protein kinase IRK	15
17.		LRR receptor-like serine/threonine-protein kinase At1g53420	15
18.		LRR receptor-like serine/threonine-protein kinase FLS2	14
19.		LRR receptor-like serine/threonine-protein kinase At4g26540	12
20.		LRR receptor-like serine/threonine-protein kinase At1g5343	10
21.		LRR receptor-like serine/threonine-protein kinase HSL2	9
22.		LRR receptor-like serine/threonine-protein kinase At2g24230	9
23.		LRR receptor-like serine/threonine-protein kinase At5g63710	9
24.		LRR receptor-like serine/threonine-protein kinase ERL1	8
25.		LRR receptor-like serine/threonine-protein kinase ERL2	8
26.		LRR receptor-like serine/threonine-protein kinase At1g3411	8
27.		LRR receptor-like serine/threonine-protein kinase At4g36180	8
28.		Leucine-rich repeat receptor-like serine/threonine/tyrosine-protein kinase SOBIR1	8
29.		LRR receptor-like serine/threonine-protein kinase At1g05700	7
30.		LRR receptor-like serine/threonine-protein kinase At4g20940	7
31.		LRR receptor-like serine/threonine-protein kinase RKF3	7
32.		LRR receptor-like serine/threonine-protein kinase RCH1	7
33.		LRR receptor-like serine/threonine-protein kinase FEI 2	6
34.		LRR receptor-like serine/threonine-protein kinase MRH1	6
35.		LRR receptor-like serine/threonine-protein kinase At1g12460	6
36.		LRR receptor-like serine/threonine-protein kinase At5g65240	6
37.		Leucine-rich repeat receptor-like serine/threonine-protein kinase At2g24130	6
38.		Receptor-like serine/threonine-protein kinase At2g40270	6
39.		Protein NSP-INTERACTING KINASE 1	5
40.		LRR receptor-like serine/threonine-protein kinase GSO2	5
41.		LRR receptor-like serine/threonine-protein kinase RPK1	5

42.		LRR receptor-like serine/threonine-protein kinase At4g37250	5
43.		LRR receptor-like serine/threonine-protein kinase At5g45780	5
44.		LRR receptor-like serine/threonine-protein kinase At2g23950	4
45.		LRR receptor-like serine/threonine-protein kinase At5g4584	4
46.		Protein NSP-INTERACTING KINASE 2	3
47.		Protein NSP-INTERACTING KINASE 3	3
48.		LRR receptor-like serine/threonine-protein kinase At1g14390	3
49.		LRR receptor-like serine/threonine-protein kinase At1g29720	3
50.		LRR receptor-like serine/threonine-protein kinase At4g2918	3
51.		LRR receptor-like serine/threonine-protein kinase At5g37450	3
52.		Leucine-rich repeat receptor-like serine/threonine-protein kinase At3g53590	3
53.		LRR receptor-like serine/threonine-protein kinase At2g0278	2
54.		LRR receptor-like serine/threonine-protein kinase At4g3052	2
55.		LRR receptor-like serine/threonine-protein kinase At4g31250	2
56.		Leucine-rich repeat receptor-like serine/threonine-protein kinase At2g14440	1
57.		Leucine-rich repeat receptor-like serine/threonine-protein kinase At1g17230	1
58.		LRR receptor-like serine/threonine-protein kinase At1g07550	1
59.		LRR receptor-like serine/threonine-protein kinase At1g51810	1
60.		LRR receptor-like serine/threonine-protein kinase At1g51880	1
61.		LRR receptor-like serine/threonine-protein kinase At5g48740	1
62.		Leucine-rich repeat receptor-like serine/threonine-protein kinase At2g04300	1
63.		G-type lectin S-receptor-like serine/threonine-protein kinase At1g61500	1
64.		Leucine-rich repeat receptor-like serine/threonine-protein kinase At3g14840	78
65.		LRR receptor-like serine/threonine-protein kinase At3g47570	58
66.		LRR receptor-like serine/threonine-protein kinase At1g56140	47
67.		LRR receptor-like serine/threonine-protein kinase FEI 1	24
68.		LRR receptor-like serine/threonine-protein kinase At5g10290	23
69.		LRR receptor-like serine/threonine-protein kinase At1g67720	22
70.	LRR-RLK (non-serine/ threonine)	Receptor-like protein kinase HSL1	41
71.		Receptor-like protein 12	39
72.		Receptor-like protein kinase HAIKU2	36
73.		Leucine-rich repeat receptor-like protein kinase At3g03770	29
74.		Leucine-rich repeat receptor-like protein kinase At5g49770	26
75.		leucine-rich repeat receptor-like protein kinase At1g35710;	20
76.		Leucine-rich repeat receptor-like protein kinase At1g66830	17
77.		Leucine-rich repeat receptor-like protein kinase At5g48380	16
78.		Leucine-rich repeat receptor-like protein kinase At3g28040	15
79.		Receptor-like protein kinase At3g47110	15
80.		Leucine-rich repeat receptor-like protein kinase At2g33170	14
81.		Leucine-rich repeat receptor-like protein kinase At2g25790	13
82.		Leucine-rich repeat receptor-like protein kinase At5g63930	13
83.		Phytosulfokine receptor 2	12
84.		Receptor-like protein kinase 5	10
85.		Leucine-rich repeat receptor-like protein kinase TDR	9
86.		Leucine-rich repeat receptor-like protein kinase At5g06940	8
87.		Leucine-rich repeat receptor-like tyrosine-protein kinase PXC3	7
88.		Leucine-rich repeat receptor-like protein kinase PXL2	7
89.		Leucine-rich repeat receptor-like protein CLAVATA2	7
90.		Receptor-like protein kinase 2	6
91.		Leucine-rich repeat receptor-like protein kinase CORYNE	5
92.		Leucine-rich repeat receptor-like protein kinase IMK2	5
93.		Leucine-rich repeat receptor-like kinase protein FLORAL ORGAN NUMBER1	4
94.		Receptor-like kinase TMK4	4
95.		Leucine-rich repeat receptor-like protein kinase PXC2	3
96.		Receptor-like kinase TMK3	3
97.		Leucine-rich repeat receptor-like protein kinase PEPR1	2
98.		Receptor-like protein kinase BRI1-like 3	2

99.		Receptor-like protein 2	2
100.		Leucine-rich repeat receptor-like kinase protein THICK TASSEL DWARF1	2
101.		Leucine-rich repeat receptor-like protein kinase IMK	1
102.		Leucine-rich repeat receptor-like protein kinase PEPR2	1
103.		Leucine-rich repeat receptor-like protein kinase PXC1	1
104.		Leucine-rich repeat receptor-like protein kinase PXL1	1
105.		Leucine-rich repeat receptor-like protein kinase At1g68400	1
106.		Leucine-rich repeat receptor-like protein kinase At2g19210	1
107.		Receptor-like protein kinase At3g21340	1
108.		Receptor-like protein kinase	1
109.	LRR-RK	Receptor protein kinase CLAVATA1	17
110.		Serine/threonine-protein kinase BRI1-like	15
111.		Leucine-rich repeat receptor protein kinase EMS1	14
112.		Receptor kinase At4g2374	12
113.		Somatic embryogenesis receptor kinase 1	12
114.		Serine/threonine-protein kinase BRI1-like 1	8
115.		Serine/threonine-protein kinase BRI1-like 2	7
116.		Receptor protein kinase TMK1	7
117.		Brassinosteroid LRR receptor kinase	4
118.		Receptor kinase At3g0288	4
119.		Receptor protein kinase-like protein At4g34220	3
120.		Receptor kinase At5g10020	3
121.		Receptor kinase At5g53320	3
122.		Receptor kinase At5g16590	3
123.		Receptor kinase At3g08680	3
124.		Receptor kinase At5g67200	3
125.		receptor kinase At5g58300	3
126.		Leucine-rich repeat receptor protein kinase MSL1	2
127.		Somatic embryogenesis receptor kinase 2	2
128.		Receptor kinase At2g26730	2
129.		Lectin-domain containing receptor kinase VI.4	1
130.		receptor kinase At1g27190	1
131.		Somatic embryogenesis receptor kinase 4	1

132.	Disease resistance/susceptibility	Disease resistance RPP13-like protein 1	86
133.		Disease resistance protein RPM1	57
134.		Disease resistance protein At3g14460	40
135.		Disease resistance protein At4g27190	28
136.		Probable disease resistance protein At4g2722	19
137.		Disease resistance protein RPS2	15
138.		Disease resistance protein At5g63020	14
139.		Disease resistance protein At1g50180	13
140.		Disease resistance protein RPS5	11
141.		Disease resistance protein At1g12280	11
142.		Disease resistance RPP13-like protein 4	9
143.		Disease resistance RPP8-like protein 2	8
144.		Disease resistance protein RPP8	8
145.		Disease resistance protein RPS6	8
146.		Disease resistance protein RFL1	7
147.		Disease resistance protein At4g19050	7
148.		Disease resistance protein At5g6691	7
149.		Disease resistance protein RGA3	7
150.		Disease resistance protein RPP13	5
151.		Disease resistance protein RGA2	5
152.		Disease resistance protein At1g61180	5
153.		Disease resistance protein At1g6131	4
154.		Disease resistance protein At1g12290	4
155.		Disease resistance protein At1g15890	4
156.		Disease resistance protein RPP4	3
157.		Disease resistance RPP13-like protein 3	3
158.		Disease resistance protein At1g59780	3
159.		Disease resistance protein At5g66900	3
160.		Disease resistance protein RDL6	3
161.		Disease resistance protein At1g5266	3
162.		Disease resistance protein At1g58390	3
163.		Disease resistance protein RPS4	3

164.		Disease resistance protein TAO1	2
165.		Disease resistance-like protein CSA1	2
166.		Disease resistance protein RPP1	2
167.		Disease resistance protein RGA1	2
168.		Disease resistance protein RGA4	2
169.		Disease resistance RPP8-like protein 4	2
170.		Disease resistance protein At1g61300	2
171.		Disease resistance protein At1g63350	2
172.		Disease resistance protein At5g43740	2
173.		Disease resistance protein At5g47250	2
174.		Disease susceptibility protein LOV1	2
175.		Disease resistance protein At1g62630	1
176.		Disease resistance protein At1g61190	1
177.		Disease resistance protein At4g10780	1
178.		Disease resistance protein At5g43730	1
179.		Disease resistance protein At1g58602	1
180.		Disease resistance RPP13-like protein 2	1
181.		Disease resistance protein RML1B	1
182.		Disease resistance RPP8-like protein 3	1
183.		Disease resistance protein RXW24L	1
184.		Disease resistance protein At4g11170	1
185.		TMV resistance protein N	1
186.		Phospholipase A I	2
187.	F-box	EIN3-binding F-box protein 1	30
188.		EIN3-binding F-box protein 2	8
189.		F-box protein FBW2	10
190.		F-box protein SKIP2	8
191.		F-box protein SKIP2	7
192.		F-box/LRR-repeat protein 15	31
193.		F-box/LRR-repeat protein 3	20
194.		F-box/LRR-repeat protein 4	13
195.		F-box protein FBX14	10

196.		F-box/LRR-repeat protein At4g29420	10
197.		F-box/FBD/LRR-repeat protein At1g13570	10
198.		F-box/LRR-repeat protein 10	8
199.		F-box/LRR-repeat MAX2 homolog A	5
200.		F-box protein At5g07670	5
201.		F-box protein At5g51380	5
202.		F-box protein At3g58530	5
203.		F-box/LRR-repeat protein At3g59200	5
204.		F-box protein At1g47056	4
205.		F-box/LRR-repeat protein 14	4
206.		F-box/LRR-repeat protein At5g63520	4
207.		F-box protein SKIP1	3
208.		F-box/LRR-repeat protein At1g67190	3
209.		F-box/LRR-repeat protein At3g48880	3
210.		F-box/LRR-repeat protein At3g59210	3
211.		F-box/LRR-repeat protein At4g14096	3
212.		F-box/FBD/LRR-repeat protein At5g5384	3
213.		F-box protein SKP2A	2
214.		F-box protein SKIP19	2
215.		F-box protein SKIP17	2
216.		F-box protein At5g67140	2
217.		F-box protein At1g58310	2
218.		F-box/LRR-repeat protein 23	2
219.		F-box/LRR-repeat protein At3g26922	2
220.		F-box/LRR-repeat protein At5g02910	2
221.		F-box/LRR-repeat protein 8	2
222.		F-box/FBD/LRR-repeat protein At2g04230	2
223.		F-box/FBD/LRR-repeat protein At5g56420	2
224.		F-box protein SKIP14	1
225.		F-box protein SKIP28	1
226.		F-box protein At4g05475	1
227.		F-box protein At1g19070	1

228.		FBD-associated F-box protein At5g3859	1
229.		FBD-associated F-box protein At5g60610	1
230.		F-box/LRR-repeat protein 12	1
231.		F-box/LRR-repeat protein 17	1
232.		F-box/LRR-repeat protein 21	1
233.		F-box/LRR-repeat protein At1g55660	1
234.		F-box/LRR-repeat protein At3g59160	1
235.		F-box/LRR-repeat protein At3g59250	1
236.		F-box/FBD/LRR-repeat protein At3g49030	1
237.		F-box/FBD/LRR-repeat protein At5g22660	1
238.		F-box/FBD/LRR-repeat protein At3g49480	1
239.		F-box/FBD/LRR-repeat protein At3g59240	1
240.		F-box/FBD/LRR-repeat protein At4g13965	1
241.		Coronatine-insensitive protein 1	2
242.	Other LRR-containing protein	Plant intracellular Ras-group-related LRR protein 6	14
243.		Plant intracellular Ras-group-related LRR protein 5	12
244.		Plant intracellular Ras-group-related LRR protein 9	6
245.		Plant intracellular Ras-group-related LRR protein 1	5
246.		Plant intracellular Ras-group-related LRR protein 4	5
247.		Plant intracellular Ras-group-related LRR protein 7	5
248.		Plant intracellular Ras-group-related LRR protein 3	4
249.		Plant intracellular Ras-group-related LRR protein 8	1
250.		Protein TRANSPORT INHIBITOR RESPONSE 1	27
251.		Protein BRASSINOSTEROID INSENSITIVE 1	21
252.		Protein STRUBBELIG-RECEPTOR FAMILY	13
253.		Leucine-rich repeat extensin-like protein 4	10
254.		Transport inhibitor response 1-like protein Os04g0395600	8
255.		RAN GTPase-activating protein 1	7
256.		Phytosulfokine receptor 1	6
257.		Tyrosine-sulphated glycopeptide receptor 1	5
258.		Transport inhibitor response 1-like protein Os04g0395600	3
259.		Leucine-rich repeat extensin-like protein 7	2

260.		Leucine-rich repeat protein soc-2 homolog	2
261.		Leucine-rich repeat extensin-like protein 2	1
262.		Proline-rich receptor-like protein kinase PERK12	1
263.		Leucine-rich repeat protein soc-2	1
264.		RAN GTPase-activating protein 2	1
265.		Protein VARIATION IN COMPOUND TRIGGERED ROOT growth response	1
266.		Tubulin-folding cofactor E	2
267.		Protein phosphatase 1 regulatory subunit SDS22 homolog	4
268.		187-kDa microtubule-associated protein AIR9	20
269.		Polygalacturonase inhibitor	8
270.		DNA-damage-repair/toleration protein DRT100	11
271.		Ribonuclease H protein At1g65750	1
272.		U2 small nuclear ribonucleoprotein A	10
273.		Mediator of RNA polymerase II transcription subunit 13	2
274.		LRR repeats and ubiquitin-like domain-containing protein At2g30105	3
275.		Cytoplasmic 60S subunit biogenesis factor REI1 homolog 1	2
276.		Cytoplasmic 60S subunit biogenesis factor REI1 homolog 2	1
277.		kinase-like protein TMKL1	1
278.	Unclear/unknown	Uncharacterised protein At4g06744	2
		Total	2389

Table S2: Pentatricopeptide repeat-containing proteins identified in the calyx transcriptome of roselle.

No.	Protein group	Protein	Number of transcripts
1.	Pentatricopeptide repeat-containing protein (*Arabidopsis* homologs)	Pentatricopeptide repeat-containing protein At1g01970	2
2.		Pentatricopeptide repeat-containing protein At1g02060	5
3.		Pentatricopeptide repeat-containing protein At1g02150	4
4.		Pentatricopeptide repeat-containing protein At1g02370	5
5.		Pentatricopeptide repeat-containing protein At1g0242	4
6.		Pentatricopeptide repeat-containing protein At1g03100	4
7.		Pentatricopeptide repeat-containing protein At1g03540	4
8.		Pentatricopeptide repeat-containing protein At1g0356	7
9.		Pentatricopeptide repeat-containing protein At1g04840	6
10.		Pentatricopeptide repeat-containing protein At1g05600	2
11.		Pentatricopeptide repeat-containing protein At1g05670	10
12.		Pentatricopeptide repeat-containing protein At1g05750	6
13.		Pentatricopeptide repeat-containing protein At1g06140	4
14.		Pentatricopeptide repeat-containing protein At1g06145	2
15.		Pentatricopeptide repeat-containing protein At1g06270	6
16.		Pentatricopeptide repeat-containing protein At1g06710	11
17.		Pentatricopeptide repeat-containing protein At1g07590	1
18.		Pentatricopeptide repeat-containing protein At1g07740	3
19.		Pentatricopeptide repeat-containing protein At1g08070	12
20.		Pentatricopeptide repeat-containing protein At1g08610	6
21.		Pentatricopeptide repeat-containing protein At1g09190	2
22.		Pentatricopeptide repeat-containing protein At1g09220	1
23.		Pentatricopeptide repeat-containing protein At1g09410	1
24.		Pentatricopeptide repeat-containing protein At1g09680	7
25.		Pentatricopeptide repeat-containing protein At1g0982	3
26.		Pentatricopeptide repeat-containing protein At1g0990	20
27.		Pentatricopeptide repeat-containing protein At1g10270	7
28.		Pentatricopeptide repeat-containing protein At1g10330	2

29.		Pentatricopeptide repeat-containing protein At1g10910	2
30.		Pentatricopeptide repeat-containing protein At1g1129	10
31.		Pentatricopeptide repeat-containing protein At1g11710	3
32.		Pentatricopeptide repeat-containing protein At1g11900	2
33.		Pentatricopeptide repeat-containing protein At1g12620	3
34.		Pentatricopeptide repeat-containing protein At1g12700	19
35.		Pentatricopeptide repeat-containing protein At1g1277	4
36.		Pentatricopeptide repeat-containing protein At1g13040	7
37.		Pentatricopeptide repeat-containing protein At1g13630	5
38.		Pentatricopeptide repeat-containing protein At1g15510	9
39.		Pentatricopeptide repeat-containing protein At1g16830	3
40.		Pentatricopeptide repeat-containing protein At1g17630	1
41.		Pentatricopeptide repeat-containing protein At1g18485	1
42.		Pentatricopeptide repeat-containing protein At1g18900	5
43.		Pentatricopeptide repeat-containing protein At1g19290	8
44.		Pentatricopeptide repeat-containing protein At1g19525	6
45.		Pentatricopeptide repeat-containing protein At1g19720	15
46.		Pentatricopeptide repeat-containing protein At1g20230	11
47.		Pentatricopeptide repeat-containing protein At1g20300	4
48.		Pentatricopeptide repeat-containing protein At1g22830	2
49.		Pentatricopeptide repeat-containing protein At1g22960	10
50.		Pentatricopeptide repeat-containing protein At1g25360	7
51.		Pentatricopeptide repeat-containing protein At1g26460	10
52.		Pentatricopeptide repeat-containing protein At1g26500	5
53.		Pentatricopeptide repeat-containing protein At1g2690	1
54.		Pentatricopeptide repeat-containing protein At1g28690	4
55.		Pentatricopeptide repeat-containing protein At1g30610	6
56.		Pentatricopeptide repeat-containing protein At1g3143	3
57.		Pentatricopeptide repeat-containing protein At1g31790	2
58.		Pentatricopeptide repeat-containing protein At1g31840	5
59.		Pentatricopeptide repeat-containing protein At1g31920	11
60.		Pentatricopeptide repeat-containing protein At1g32415	4

61.		Pentatricopeptide repeat-containing protein At1g33350	11
62.		Pentatricopeptide repeat-containing protein At1g34160	6
63.		Pentatricopeptide repeat-containing protein At1g43980	4
64.		Pentatricopeptide repeat-containing protein At1g50270	2
65.		Pentatricopeptide repeat-containing protein At1g51965	14
66.		Pentatricopeptide repeat-containing protein At1g52620	2
67.		Pentatricopeptide repeat-containing protein At1g52640	3
68.		Pentatricopeptide repeat-containing protein At1g53330	3
69.		Pentatricopeptide repeat-containing protein At1g53600	5
70.		Pentatricopeptide repeat-containing protein At1g55630	3
71.		Pentatricopeptide repeat-containing protein At1g55630	2
72.		Pentatricopeptide repeat-containing protein At1g56690	3
73.		Pentatricopeptide repeat-containing protein At1g59720	6
74.		Pentatricopeptide repeat-containing protein At1g60770	3
75.		Pentatricopeptide repeat-containing protein At1g6187	9
76.		Pentatricopeptide repeat-containing protein At1g62260	3
77.		Pentatricopeptide repeat-containing protein At1g62350	3
78.		Pentatricopeptide repeat-containing protein At1g62590	1
79.		Pentatricopeptide repeat-containing protein At1g62670	19
80.		Pentatricopeptide repeat-containing protein At1g62680	1
81.		Pentatricopeptide repeat-containing protein At1g62720	5
82.		Pentatricopeptide repeat-containing protein At1g62910	2
83.		Pentatricopeptide repeat-containing protein At1g62930	26
84.		Pentatricopeptide repeat-containing protein At1g6307	2
85.		Pentatricopeptide repeat-containing protein At1g63080	5
86.		Pentatricopeptide repeat-containing protein At1g63150	6
87.		Pentatricopeptide repeat-containing protein At1g63330	1
88.		Pentatricopeptide repeat-containing protein At1g63400	4
89.		Pentatricopeptide repeat-containing protein At1g64100	1
90.		Pentatricopeptide repeat-containing protein At1g6431	1
91.		Pentatricopeptide repeat-containing protein At1g6431	1
92.		Pentatricopeptide repeat-containing protein At1g64583	1

93.		Pentatricopeptide repeat-containing protein At1g66345	4
94.		Pentatricopeptide repeat-containing protein At1g68930	6
95.		Pentatricopeptide repeat-containing protein At1g69290	8
96.		Pentatricopeptide repeat-containing protein At1g69350	4
97.		Pentatricopeptide repeat-containing protein At1g71060	8
98.		Pentatricopeptide repeat-containing protein At1g71210	20
99.		Pentatricopeptide repeat-containing protein At1g71420	5
100.		Pentatricopeptide repeat-containing protein At1g71460	3
101.		Pentatricopeptide repeat-containing protein At1g71490	7
102.		Pentatricopeptide repeat-containing protein At1g73400	6
103.		Pentatricopeptide repeat-containing protein At1g73710	20
104.		Pentatricopeptide repeat-containing protein At1g74400	3
105.		Pentatricopeptide repeat-containing protein At1g74580	5
106.		Pentatricopeptide repeat-containing protein At1g74600	3
107.		Pentatricopeptide repeat-containing protein At1g74630	4
108.		Pentatricopeptide repeat-containing protein At1g74750	4
109.		Pentatricopeptide repeat-containing protein At1g74850	6
110.		Pentatricopeptide repeat-containing protein At1g74900	5
111.		Pentatricopeptide repeat-containing protein At1g76280	7
112.		Pentatricopeptide repeat-containing protein At1g77010	2
113.		Pentatricopeptide repeat-containing protein At1g77170	3
114.		Pentatricopeptide repeat-containing protein At1g77360	9
115.		Pentatricopeptide repeat-containing protein At1g77405	3
116.		Pentatricopeptide repeat-containing protein At1g79080	9
117.		Pentatricopeptide repeat-containing protein At1g79490	7
118.		Pentatricopeptide repeat-containing protein At1g79540	7
119.		Pentatricopeptide repeat-containing protein At1g80150	7
120.		Pentatricopeptide repeat-containing protein At1g80270	8
121.		Pentatricopeptide repeat-containing protein At1g80550	3
122.		Pentatricopeptide repeat-containing protein At1g80880	2
123.		Pentatricopeptide repeat-containing protein At2g01390	2
124.		Pentatricopeptide repeat-containing protein At2g01510	9

125.		Pentatricopeptide repeat-containing protein At2g01740	4
126.		Pentatricopeptide repeat-containing protein At2g01860	3
127.		Pentatricopeptide repeat-containing protein At2g02150	5
128.		Pentatricopeptide repeat-containing protein At2g02980	4
129.		Pentatricopeptide repeat-containing protein At2g03380	3
130.		Pentatricopeptide repeat-containing protein At2g03880	5
131.		Pentatricopeptide repeat-containing protein At2g04860	1
132.		Pentatricopeptide repeat-containing protein At2g06000	12
133.		Pentatricopeptide repeat-containing protein At2g13420	6
134.		Pentatricopeptide repeat-containing protein At2g13600	10
135.		Pentatricopeptide repeat-containing protein At2g15630	3
136.		Pentatricopeptide repeat-containing protein At2g15690	16
137.		Pentatricopeptide repeat-containing protein At2g15820	6
138.		Pentatricopeptide repeat-containing protein At2g15980	3
139.		Pentatricopeptide repeat-containing protein At2g16880	4
140.		Pentatricopeptide repeat-containing protein At2g17033	3
141.		Pentatricopeptide repeat-containing protein At2g17140	5
142.		Pentatricopeptide repeat-containing protein At2g17210	2
143.		Pentatricopeptide repeat-containing protein At2g17525	2
144.		Pentatricopeptide repeat-containing protein At2g17670	4
145.		Pentatricopeptide repeat-containing protein At2g18940	5
146.		Pentatricopeptide repeat-containing protein At2g19280	6
147.		Pentatricopeptide repeat-containing protein At2g2054	1
148.		Pentatricopeptide repeat-containing protein At2g2071	3
149.		Pentatricopeptide repeat-containing protein At2g21090	6
150.		Pentatricopeptide repeat-containing protein At2g22070	6
151.		Pentatricopeptide repeat-containing protein At2g2241	2
152.		Pentatricopeptide repeat-containing protein At2g25580	2
153.		Pentatricopeptide repeat-containing protein At2g26790	5
154.		Pentatricopeptide repeat-containing protein At2g2761	4
155.		Pentatricopeptide repeat-containing protein At2g27800	5
156.		Pentatricopeptide repeat-containing protein At2g28050	1

157.		Pentatricopeptide repeat-containing protein At2g29760	5
158.		Pentatricopeptide repeat-containing protein At2g30100	12
159.		Pentatricopeptide repeat-containing protein At2g30780	9
160.		Pentatricopeptide repeat-containing protein At2g31400	7
161.		Pentatricopeptide repeat-containing protein At2g32630	5
162.		Pentatricopeptide repeat-containing protein At2g33680	6
163.		Pentatricopeptide repeat-containing protein At2g33760	4
164.		Pentatricopeptide repeat-containing protein At2g34400	10
165.		Pentatricopeptide repeat-containing protein At2g35030	13
166.		Pentatricopeptide repeat-containing protein At2g35130	9
167.		Pentatricopeptide repeat-containing protein At2g36240	6
168.		Pentatricopeptide repeat-containing protein At2g36730	4
169.		Pentatricopeptide repeat-containing protein At2g36980	2
170.		Pentatricopeptide repeat-containing protein At2g37230	5
171.		Pentatricopeptide repeat-containing protein At2g37310	3
172.		Pentatricopeptide repeat-containing protein At2g37320	2
173.		Pentatricopeptide repeat-containing protein At2g38420	2
174.		Pentatricopeptide repeat-containing protein At2g3923	8
175.		Pentatricopeptide repeat-containing protein At2g39620	1
176.		Pentatricopeptide repeat-containing protein At2g40240	2
177.		Pentatricopeptide repeat-containing protein At2g40720	6
178.		Pentatricopeptide repeat-containing protein At2g41080	4
179.		Pentatricopeptide repeat-containing protein At2g4172	5
180.		Pentatricopeptide repeat-containing protein At2g42920	6
181.		Pentatricopeptide repeat-containing protein At2g44880	2
182.		Pentatricopeptide repeat-containing protein At2g45350	8
183.		Pentatricopeptide repeat-containing protein At2g48000	1
184.		Pentatricopeptide repeat-containing protein At3g0158	2
185.		Pentatricopeptide repeat-containing protein At3g0233	5
186.		Pentatricopeptide repeat-containing protein At3g02490	3
187.		Pentatricopeptide repeat-containing protein At3g0265	8
188.		Pentatricopeptide repeat-containing protein At3g03580	7

189.		Pentatricopeptide repeat-containing protein At3g04130	4
190.		Pentatricopeptide repeat-containing protein At3g04750	1
191.		Pentatricopeptide repeat-containing protein At3g04760	5
192.		Pentatricopeptide repeat-containing protein At3g0534	3
193.		Pentatricopeptide repeat-containing protein At3g0643	7
194.		Pentatricopeptide repeat-containing protein At3g06920	4
195.		Pentatricopeptide repeat-containing protein At3g07290	7
196.		Pentatricopeptide repeat-containing protein At3g08820	4
197.		Pentatricopeptide repeat-containing protein At3g09040	3
198.		Pentatricopeptide repeat-containing protein At3g09060	3
199.		Pentatricopeptide repeat-containing protein At3g09650	6
200.		Pentatricopeptide repeat-containing protein At3g11460	5
201.		Pentatricopeptide repeat-containing protein At3g12770	6
202.		Pentatricopeptide repeat-containing protein At3g13150	7
203.		Pentatricopeptide repeat-containing protein At3g13160	2
204.		Pentatricopeptide repeat-containing protein At3g13770	6
205.		Pentatricopeptide repeat-containing protein At3g13880	5
206.		Pentatricopeptide repeat-containing protein At3g14330	3
207.		Pentatricopeptide repeat-containing protein At3g14580	1
208.		Pentatricopeptide repeat-containing protein At3g14730	5
209.		Pentatricopeptide repeat-containing protein At3g15130	5
210.		Pentatricopeptide repeat-containing protein At3g15200	2
211.		Pentatricopeptide repeat-containing protein At3g15930	2
212.		Pentatricopeptide repeat-containing protein At3g16010	9
213.		Pentatricopeptide repeat-containing protein At3g16010	1
214.		Pentatricopeptide repeat-containing protein At3g16710	2
215.		Pentatricopeptide repeat-containing protein At3g16890	3
216.		Pentatricopeptide repeat-containing protein At3g18110	6
217.		Pentatricopeptide repeat-containing protein At3g18840	2
218.		Pentatricopeptide repeat-containing protein At3g18970	3
219.		Pentatricopeptide repeat-containing protein At3g20730	4
220.		Pentatricopeptide repeat-containing protein At3g21470	5

221.		Pentatricopeptide repeat-containing protein At3g22150	8
222.		Pentatricopeptide repeat-containing protein At3g22470	3
223.		Pentatricopeptide repeat-containing protein At3g22670	3
224.		Pentatricopeptide repeat-containing protein At3g22690	14
225.		Pentatricopeptide repeat-containing protein At3g23020	5
226.		Pentatricopeptide repeat-containing protein At3g23330	2
227.		Pentatricopeptide repeat-containing protein At3g24000	4
228.		Pentatricopeptide repeat-containing protein At3g25060	1
229.		Pentatricopeptide repeat-containing protein At3g25210	5
230.		Pentatricopeptide repeat-containing protein At3g25970	5
231.		Pentatricopeptide repeat-containing protein At3g26540	6
232.		Pentatricopeptide repeat-containing protein At3g26630	5
233.		Pentatricopeptide repeat-containing protein At3g26782	7
234.		Pentatricopeptide repeat-containing protein At3g29230	12
235.		Pentatricopeptide repeat-containing protein At3g29290	3
236.		Pentatricopeptide repeat-containing protein At3g42630	2
237.		Pentatricopeptide repeat-containing protein At3g46610	4
238.		Pentatricopeptide repeat-containing protein At3g46790	6
239.		Pentatricopeptide repeat-containing protein At3g46870	3
240.		Pentatricopeptide repeat-containing protein At3g47530	3
241.		Pentatricopeptide repeat-containing protein At3g47840	1
242.		Pentatricopeptide repeat-containing protein At3g48250	8
243.		Pentatricopeptide repeat-containing protein At3g48810	3
244.		Pentatricopeptide repeat-containing protein At3g49142	3
245.		Pentatricopeptide repeat-containing protein At3g49170	9
246.		Pentatricopeptide repeat-containing protein At3g49240	5
247.		Pentatricopeptide repeat-containing protein At3g49710	7
248.		Pentatricopeptide repeat-containing protein At3g49730	3
249.		Pentatricopeptide repeat-containing protein At3g49740	5
250.		Pentatricopeptide repeat-containing protein At3g50420	6
251.		Pentatricopeptide repeat-containing protein At3g53170	3
252.		Pentatricopeptide repeat-containing protein At3g53700	2

253.		Pentatricopeptide repeat-containing protein At3g54980	3
254.		Pentatricopeptide repeat-containing protein At3g57430	7
255.		Pentatricopeptide repeat-containing protein At3g58590	4
256.		Pentatricopeptide repeat-containing protein At3g59040	11
257.		Pentatricopeptide repeat-containing protein At3g60050	1
258.		Pentatricopeptide repeat-containing protein At3g61360	7
259.		Pentatricopeptide repeat-containing protein At3g61520	5
260.		Pentatricopeptide repeat-containing protein At3g62470	3
261.		Pentatricopeptide repeat-containing protein At3g6289	7
262.		Pentatricopeptide repeat-containing protein At3g63370	5
263.		Pentatricopeptide repeat-containing protein At4g01400	3
264.		Pentatricopeptide repeat-containing protein At4g01570	6
265.		Pentatricopeptide repeat-containing protein At4g02750	11
266.		Pentatricopeptide repeat-containing protein At4g02820	8
267.		Pentatricopeptide repeat-containing protein At4g0437	4
268.		Pentatricopeptide repeat-containing protein At4g04790	6
269.		Pentatricopeptide repeat-containing protein At4g11690	7
270.		Pentatricopeptide repeat-containing protein At4g13650	4
271.		Pentatricopeptide repeat-containing protein At4g14050	5
272.		Pentatricopeptide repeat-containing protein At4g1419	3
273.		Pentatricopeptide repeat-containing protein At4g14820	6
274.		Pentatricopeptide repeat-containing protein At4g14850	2
275.		Pentatricopeptide repeat-containing protein At4g16390	7
276.		Pentatricopeptide repeat-containing protein At4g16470	3
277.		Pentatricopeptide repeat-containing protein At4g16835	5
278.		Pentatricopeptide repeat-containing protein At4g17616	1
279.		Pentatricopeptide repeat-containing protein At4g17915	4
280.		Pentatricopeptide repeat-containing protein At4g18520	4
281.		Pentatricopeptide repeat-containing protein At4g18840	11
282.		Pentatricopeptide repeat-containing protein At4g18975	6
283.		Pentatricopeptide repeat-containing protein At4g19191	1
284.		Pentatricopeptide repeat-containing protein At4g19440	7

285.		Pentatricopeptide repeat-containing protein At4g19890	6
286.		Pentatricopeptide repeat-containing protein At4g20090	4
287.		Pentatricopeptide repeat-containing protein At4g20740	8
288.		Pentatricopeptide repeat-containing protein At4g20770	4
289.		Pentatricopeptide repeat-containing protein At4g21065	8
290.		Pentatricopeptide repeat-containing protein At4g21170	3
291.		Pentatricopeptide repeat-containing protein At4g21190	11
292.		Pentatricopeptide repeat-containing protein At4g21300	6
293.		Pentatricopeptide repeat-containing protein At4g21705	6
294.		Pentatricopeptide repeat-containing protein At4g21880	1
295.		Pentatricopeptide repeat-containing protein At4g2276	5
296.		Pentatricopeptide repeat-containing protein At4g25270	3
297.		Pentatricopeptide repeat-containing protein At4g26680	3
298.		Pentatricopeptide repeat-containing protein At4g28010	2
299.		Pentatricopeptide repeat-containing protein At4g30700	12
300.		Pentatricopeptide repeat-containing protein At4g3082	5
301.		Pentatricopeptide repeat-containing protein At4g31850	5
302.		Pentatricopeptide repeat-containing protein At4g32430	6
303.		Pentatricopeptide repeat-containing protein At4g32450	2
304.		Pentatricopeptide repeat-containing protein At4g33170	3
305.		Pentatricopeptide repeat-containing protein At4g33990	5
306.		Pentatricopeptide repeat-containing protein At4g3513	6
307.		Pentatricopeptide repeat-containing protein At4g35850	5
308.		Pentatricopeptide repeat-containing protein At4g36680	9
309.		Pentatricopeptide repeat-containing protein At4g37170	6
310.		Pentatricopeptide repeat-containing protein At4g38150	3
311.		Pentatricopeptide repeat-containing protein At4g39530	1
312.		Pentatricopeptide repeat-containing protein At4g39620	3
313.		Pentatricopeptide repeat-containing protein At4g39952	5
314.		Pentatricopeptide repeat-containing protein At5g01110	4
315.		Pentatricopeptide repeat-containing protein At5g0283	8
316.		Pentatricopeptide repeat-containing protein At5g02860	7

317.		Pentatricopeptide repeat-containing protein At5g03800	4
318.		Pentatricopeptide repeat-containing protein At5g0478	2
319.		Pentatricopeptide repeat-containing protein At5g04810	13
320.		Pentatricopeptide repeat-containing protein At5g06400	4
321.		Pentatricopeptide repeat-containing protein At5g06540	6
322.		Pentatricopeptide repeat-containing protein At5g08305	3
323.		Pentatricopeptide repeat-containing protein At5g08310	8
324.		Pentatricopeptide repeat-containing protein At5g08490	3
325.		Pentatricopeptide repeat-containing protein At5g08510	6
326.		Pentatricopeptide repeat-containing protein At5g09450	3
327.		Pentatricopeptide repeat-containing protein At5g09950	4
328.		Pentatricopeptide repeat-containing protein At5g10690	7
329.		Pentatricopeptide repeat-containing protein At5g11310	3
330.		Pentatricopeptide repeat-containing protein At5g12100	5
331.		Pentatricopeptide repeat-containing protein At5g13230	1
332.		Pentatricopeptide repeat-containing protein At5g13230	1
333.		Pentatricopeptide repeat-containing protein At5g13270	5
334.		Pentatricopeptide repeat-containing protein At5g13770	5
335.		Pentatricopeptide repeat-containing protein At5g14080	2
336.		Pentatricopeptide repeat-containing protein At5g14770	11
337.		Pentatricopeptide repeat-containing protein At5g15010	3
338.		Pentatricopeptide repeat-containing protein At5g15300	7
339.		Pentatricopeptide repeat-containing protein At5g15340	3
340.		Pentatricopeptide repeat-containing protein At5g1642	3
341.		Pentatricopeptide repeat-containing protein At5g1664	5
342.		Pentatricopeptide repeat-containing protein At5g16860	4
343.		Pentatricopeptide repeat-containing protein At5g18390	4
344.		Pentatricopeptide repeat-containing protein At5g18475	6
345.		Pentatricopeptide repeat-containing protein At5g18950	4
346.		Pentatricopeptide repeat-containing protein At5g19020	5
347.		Pentatricopeptide repeat-containing protein At5g21222	3
348.		Pentatricopeptide repeat-containing protein At5g25630	3

349.		Pentatricopeptide repeat-containing protein At5g27110	1
350.		Pentatricopeptide repeat-containing protein At5g27270	6
351.		Pentatricopeptide repeat-containing protein At5g27460	2
352.		Pentatricopeptide repeat-containing protein At5g28460	7
353.		Pentatricopeptide repeat-containing protein At5g36300	1
354.		Pentatricopeptide repeat-containing protein At5g37570	9
355.		Pentatricopeptide repeat-containing protein At5g38730	8
356.		Pentatricopeptide repeat-containing protein At5g39350	3
357.		Pentatricopeptide repeat-containing protein At5g39680	2
358.		Pentatricopeptide repeat-containing protein At5g39710	7
359.		Pentatricopeptide repeat-containing protein At5g39980	6
360.		Pentatricopeptide repeat-containing protein At5g40400	1
361.		Pentatricopeptide repeat-containing protein At5g40405	2
362.		Pentatricopeptide repeat-containing protein At5g4041	4
363.		Pentatricopeptide repeat-containing protein At5g41170	1
364.		Pentatricopeptide repeat-containing protein At5g42310	6
365.		Pentatricopeptide repeat-containing protein At5g43790	7
366.		Pentatricopeptide repeat-containing protein At5g43820	6
367.		Pentatricopeptide repeat-containing protein At5g44230	7
368.		Pentatricopeptide repeat-containing protein At5g46100	7
369.		Pentatricopeptide repeat-containing protein At5g46460	2
370.		Pentatricopeptide repeat-containing protein At5g46580	8
371.		Pentatricopeptide repeat-containing protein At5g47360	6
372.		Pentatricopeptide repeat-containing protein At5g48730	5
373.		Pentatricopeptide repeat-containing protein At5g48910	9
374.		Pentatricopeptide repeat-containing protein At5g50280	8
375.		Pentatricopeptide repeat-containing protein At5g50390	5
376.		Pentatricopeptide repeat-containing protein At5g5099	5
377.		Pentatricopeptide repeat-containing protein At5g52630	3
378.		Pentatricopeptide repeat-containing protein At5g52850	4
379.		Pentatricopeptide repeat-containing protein At5g55740	6
380.		Pentatricopeptide repeat-containing protein At5g55840	5

381.		Pentatricopeptide repeat-containing protein At5g56310	1
382.		Pentatricopeptide repeat-containing protein At5g57250	9
383.		Pentatricopeptide repeat-containing protein At5g59200	7
384.		Pentatricopeptide repeat-containing protein At5g59600	2
385.		Pentatricopeptide repeat-containing protein At5g59900	6
386.		Pentatricopeptide repeat-containing protein At5g61370	6
387.		Pentatricopeptide repeat-containing protein At5g6140	3
388.		Pentatricopeptide repeat-containing protein At5g61990	10
389.		Pentatricopeptide repeat-containing protein At5g64320	6
390.		Pentatricopeptide repeat-containing protein At5g65560	7
391.		Pentatricopeptide repeat-containing protein At5g65820	1
392.		Pentatricopeptide repeat-containing protein At5g66500	1
393.		Pentatricopeptide repeat-containing protein At5g66520	9
394.		Pentatricopeptide repeat-containing protein At5g66631	3
395.		Pentatricopeptide repeat-containing protein At5g67570	9
396.	Known tetratricopeptide repeat-containing proteins	Pentatricopeptide repeat-containing protein MRL1	12
397.		Pentatricopeptide repeat protein for germination on NaCl (PGN)	8
398.		Protein NUCLEAR FUSION DEFECTIVE 5	5
399.		Reticulon-like protein B22	6
400.		Pentatricopeptide repeat-containing protein DOT4	7
401.		Pentatricopeptide repeat-containing protein ELI1	6
402.		Transcription factor bHLH15	2
403.		Transcription factor EMB1444	9
404.		Proteinaceous RNase P 1, chloroplastic/mitochondrial	2
405.		Pentatricopeptide repeat-containing protein PNM	2
406.		Pentatricopeptide repeat-containing protein OTP51	1
407.	Unclear/unknown	No match	16
		Total	2091

Table S3: WD40 repeat-containing proteins identified in the calyx transcriptome of roselle.

No.	Protein group	Protein	Number of transcripts
1.	Katanin	Katanin p80 WD40 repeat-containing subunit B1 homolog {ECO:0000255|HAMAP-Rule:MF_03022}	38
2.	Topless-related protein	Topless-related protein 1	11
3.		Topless-related protein 2	8
4.		Topless-related protein 3	9
5.		Topless-related protein 4	8
6.		Protein TOPLESS	3
7.	Protein MSI	WD-40 repeat-containing protein MSI1	5
8.		WD-40 repeat-containing protein MSI2	3
9.		WD-40 repeat-containing protein MSI4	21
10.		WD-40 repeat-containing protein MSI5	3
11.	Autophagy-related	Autophagy-related protein 16 {ECO:0000305}	5
12.		Autophagy-related protein 18a	12
13.		Autophagy-related protein 18c	4
14.		Autophagy-related protein 18d	3
15.		Autophagy-related protein 18f	4
16.		Autophagy-related protein 18g	2
17.		Autophagy-related protein 18h	1
18.	Transcriptional-related	Transcription initiation factor TFIID subunit 5	11
19.		Transcriptional corepressor LEUNIG {ECO:0000303|PubMed:11058164	10
20.		Transcriptional corepressor LEUNIG_HOMOLOG	5
21.		Zinc finger CCCH domain-containing protein 17	1
22.		Zinc finger CCCH domain-containing protein 48	1
23.		Zinc finger CCCH domain-containing protein 62	2
24.		Zinc finger CCCH domain-containing protein 63	5
25.	RNA processing proteins	Pre-mRNA-processing factor 19 {ECO:0000305}	5
26.		Pre-mRNA-processing factor 19 homolog 1 {ECO:0000305}	1
27.		Pre-mRNA-processing factor 171	1
28.	Ribosome biogenesis	U3 small nucleolar RNA-associated protein 18 homolog	5
29.		U3 small nucleolar RNA-associated protein 21 homolog	3
30.		U3 small nucleolar RNA-associated protein 13	1
31.		U3 snoRNP-associated protein-like YAO {ECO:0000305}	4
32.		U3 snoRNP-associated protein-like EMB2271 {ECO:0000305}	1
33.		U3 small nucleolar RNA-associated protein 4	2
34.		Probable U3 small nucleolar RNA-associated protein 7	2
35.		U4/U6 small nuclear ribonucleoprotein PRP4-like protein	1
36.		Ribosome biogenesis protein BOP1 homolog {ECO:0000255|HAMAP-Rule:MF_03027}	5
37.		Ribosome biogenesis protein bop1-A {ECO:0000255|HAMAP-Rule:MF_03027}	1
38.	Cell cycle regulation	Protein FIZZY-RELATED 1	2
39.		Protein FIZZY-RELATED 2	8
40.		Protein FIZZY-RELATED 3	1
41.		Cell division cycle 20.2, cofactor of APC complex	1
42.		Cell division cycle 20.1, cofactor of APC complex	1
43.		Anaphase-promoting complex subunit 4	2
44.		Mitotic checkpoint protein BUB3.2	2
45.		Mitotic checkpoint protein BUB3.1	1
46.	Other WD repeat-containing protein	LEC14B homolog/protein	14
47.		DENN domain and WD repeat-containing protein SCD1 {ECO:0000305}	14
48.		Peptidyl-prolyl cis-trans isomerase CYP21-4	11

49.		Enhancer of mRNA-decapping protein 4	10
50.		WD repeat-containing protein LWD1	9
51.		Protein pleiotropic regulatory locus 1	9
52.		SEC12-like protein 2	8
53.		Elongator complex protein 2	7
54.		Nuclear pore complex protein NUP43 {ECO:0000303|PubMed:21189294	7
55.		Protein transport protein SEC13 homolog B	7
56.		Serine/threonine protein phosphatase 2A 55 kDa regulatory subunit B beta isoform	6
57.		Guanine nucleotide-binding protein subunit beta-like protein	6
58.		Protein transport protein SEC31 homolog B	6
59.		WD repeat-containing protein 55	6
60.		Peptidyl-prolyl cis-trans isomerase CYP71	5
61.		Protein CIA1 {ECO:0000303|PubMed:23104832}	4
62.		Protein Asterix	4
63.		Eukaryotic translation initiation factor 3 subunit I {ECO:0000255|HAMAP-Rule:MF_03008}	4
64.		Guanine nucleotide-binding protein subunit beta-2	4
65.		Notchless protein homolog	4
66.		Peroxisome biogenesis protein 7	4
67.		Protein SPA1-RELATED 2	4
68.		66 kDa stress protein	4
69.		Protein SLOW WALKER 1 {ECO:0000303|PubMed:15980260}	3
70.		F-box/WD-40 repeat-containing protein At5g21040	3
71.		Guanine nucleotide-binding protein subunit beta-1	3
72.		Putative E3 ubiquitin-protein ligase LIN-1 {ECO:0000250| UniProtKB:D1FP53}	3
73.		SEC12-like protein 1	3
74.		WD repeat-containing protein RUP2	3
75.		Suppressor of mec-8 and unc-52 protein homolog 1	3
76.		Protein SUPPRESSOR OF PHYA-105 1	3
77.		WD repeat-containing protein VIP3 {ECO:0000305}	3
78.		COMPASS-like H3K4 histone methylase component WDR5B {ECO:0000303|PubMed:19567704}	3
79.		Aladin {ECO:0000303|PubMed:21189294}	2
80.		Probable glutamyl endopeptidase, chloroplastic	2
81.		E3 ubiquitin-protein ligase COP1	2
82.		Cleavage stimulation factor subunit 50 {ECO:0000303|PubMed:12379796}	2
83.		Polycomb group protein FIE2	2
84.		Protein GFS12 {ECO:0000303|PubMed:25618824}	2
85.		Protein NEDD1 {ECO:0000303|PubMed:19383896}	2
86.		Protein ROOT INITIATION DEFECTIVE 3	2
87.		Periodic tryptophan protein 1 homolog	2
88.		Periodic tryptophan protein 2 homolog	2
89.		Protein RAE1 {ECO:0000303|PubMed:21189294}	2
90.		Probable E3 ubiquitin ligase complex SCF subunit sconB	2
91.		WD repeat-containing protein wat1	2
92.		COMPASS-like H3K4 histone methylase component WDR5A	1
93.		Serine/threonine protein phosphatase 2A 55 kDa regulatory subunit B alpha isoform	1
94.		Actin-related protein 2/3 complex subunit 1B	1
95.		Protein SPIRRIG {ECO:0000303|PubMed:19392685}	1
96.		BEACH domain-containing protein A2	1
97.		Probable cytosolic iron-sulfur protein assembly protein 1	1
98.		Chromatin assembly factor 1 subunit FAS2	1
99.		F-box/WD repeat-containing protein 7	1
100.		FIP1[V]-like protein {ECO:0000303|PubMed:16282318}	1
101.		Flowering time control protein FY	1

102.		Protein HIRA	1
103.		Protein SPA1-RELATED 3	1
104.		PHO complex subunit 3	1
105.		Protein TRANSPARENT TESTA GLABRA 1	1
106.	Unclear/unknown	Uncharacterised WD-repeat containing protein	29
107.		No match	48
		Total	529

Table S4: Ankyrin repeat-containing proteins identified in the calyx transcriptome of roselle.

No.	Protein group	Protein	Number of transcripts
1.	ADP-ribosylation factor GTPase-activating protein	ADP-ribosylation factor GTPase-activating protein AGD1	17
2.		ADP-ribosylation factor GTPase-activating protein AGD2	11
3.		ADP-ribosylation factor GTPase-activating protein AGD3	18
4.		ADP-ribosylation factor GTPase-activating protein AGD4	12
5.		ADP-ribosylation factor GTPase-activating protein AGD5	12
6.		ADP-ribosylation factor GTPase-activating protein AGD6	6
7.		ADP-ribosylation factor GTPase-activating protein AGD7	8
8.		ADP-ribosylation factor GTPase-activating protein AGD8	9
9.		ADP-ribosylation factor GTPase-activating protein AGD9	3
10.	E3 ubiquitin-protein ligase	E3 ubiquitin-protein ligase KEG	34
11.		E3 ubiquitin-protein ligase XBAT31	17
12.		E3 ubiquitin-protein ligase XBAT33	14
13.		E3 ubiquitin-protein ligase XBAT35	7
14.		E3 ubiquitin-protein ligase XBAT32	5
15.		E3 ubiquitin-protein ligase XB3	1
16.		E3 ubiquitin-protein ligase XBOS33	1
17.		E3 ubiquitin-protein ligase XBOS35	1
18.	Regulatory protein NPR	Regulatory protein NPR1	15
19.		Regulatory protein NPR2	1
20.		Regulatory protein NPR4	10
21.		Regulatory protein NPR5	4
22.	Other ANK-repeat containing protein	Protein S-acyltransferase 24	28
23.		Probable protein S-acyltransferase 23	6
24.		Ankyrin repeat protein SKIP35	16
25.		BTB/POZ domain-containing protein At2g04740	4
26.		Ankyrin repeat domain-containing protein EMB506	4
27.		Ankyrin repeat-containing protein At5g02620	37
28.		Ankyrin repeat-containing protein At3g12360	30
29.		Ankyrin repeat domain-containing protein 2B {ECO:0000303|PubMed:20215589	24
30.		Ankyrin repeat-containing protein At2g01680	16
31.		Ankyrin repeat domain-containing protein, chloroplastic	6
32.		Ankyrin repeat-containing protein P16F5.05c	1
33.		Acyl-CoA-binding domain-containing protein 1	4
34.		Acyl-CoA-binding domain-containing protein 2	1
35.		Signal recognition particle 43 kDa protein	2
36.		26S proteasome non-ATPase regulatory subunit 10	2
37.		Ankyrin repeat domain-containing protein 2A {ECO:0000303|PubMed:18193034}	2
38.		Protein ACCELERATED CELL DEATH 6 {ECO:0000303|PubMed:10488236}	1
39.		Serine/threonine-protein kinase CTR1 {ECO:0000303|PubMed:8431946}	1
40.		Potassium channel KOR1	1
41.		Dual specificity protein kinase shkC	1
42.		Putative ankyrin repeat protein FPV234	1
43.	Unclear/unknown	No match	4

Total	398

Table S5: Kelch repeat-containing proteins identified in the calyx transcriptome of roselle.

No.	Protein group	Protein	Number of transcripts
1.	F-box/Kelch-repeat SKIP protein	F-box/kelch-repeat protein SKIP4	3
2.		F-box/kelch-repeat protein SKIP6	4
3.		F-box/kelch-repeat protein SKIP11	15
4.		F-box/kelch-repeat protein SKIP25	2
5.		F-box/kelch-repeat protein SKIP30	9
6.	F-box/kelch-repeat protein (*Arabidopsis* homologs)	F-box/kelch-repeat protein At1g55270	14
7.		F-box/kelch-repeat protein At1g15670	11
8.		F-box/kelch-repeat protein At4g19870	11
9.		F-box/kelch-repeat protein At5g42350	11
10.		F-box/kelch-repeat protein At3g06240	10
11.		F-box/kelch-repeat protein At1g23390	9
12.		F-box/kelch-repeat protein At1g22040	8
13.		F-box/kelch-repeat protein At1g30090	7
14.		F-box/kelch-repeat protein At1g51550	7
15.		F-box/kelch-repeat protein At3g23880	7
16.		F-box/kelch-repeat protein At2g44130	6
17.		F-box/kelch-repeat protein At1g67480	5
18.		F-box/kelch-repeat protein At1g80440	5
19.		F-box/kelch-repeat protein At3g24760	5
20.		F-box/kelch-repeat protein At3g61590	5
21.		F-box/kelch-repeat protein At5g60570	5
22.		F-box/kelch-repeat protein At1g16250	4
23.		F-box/kelch-repeat protein At5g26960	4
24.		F-box/kelch-repeat protein At1g57790	3
25.		F-box/kelch-repeat protein At5g15710	3
26.		F-box/kelch-repeat protein At5g42360	3
27.		F-box/kelch-repeat protein At3g27150	2
28.		F-box/kelch-repeat protein OR23	4
29.		F-box/kelch-repeat protein At1g74510	1
30.		F-box/kelch-repeat protein At2g29600	1
31.		F-box/kelch-repeat protein At4g35120	1
32.		F-box/LRR-repeat/kelch-repeat protein At2g27520	1
33.	Other Kelch repeat-containing protein	Acyl-CoA-binding domain-containing protein 4 (ACPB4)	40
34.		Acyl-CoA-binding domain-containing protein 5	3
35.		Adagio protein 1	5
36.		Adagio protein 3	4
37.		Serine/threonine-protein phosphatase BSL1	5
38.		Serine/threonine-protein phosphatase BSL2	5
39.		Serine/threonine-protein phosphatase BSL3	3
40.		RING finger protein B	19
41.		F-box protein AFR	8
42.		F-box only protein 13	1
43.		BTB/POZ domain-containing protein At2g3060	4
44.		BTB/POZ domain-containing protein At2g4626	1
45.		Nitrile-specifier protein 5	3
46.		Dynein regulatory complex subunit 7	2
47.		Protein UNUSUAL FLORAL ORGANS	1
48.		RNA wybutosine-synthesising protein 2/3/4	1
49.		SKP1-interacting partner 15	1
50.		TB/POZ domain-containing protein POB1	1
51.		Kelch repeat-containing protein At3g27220	8
52.	Unclear/unknown	No match	11
		Total	312

Table S6: Tetratricopeptide repeat-containing proteins identified in the calyx transcriptome of roselle.

No.	Protein group	Protein	Number of transcripts
1.	Peptidyl-prolyl cis-trans isomerase	Peptidyl-prolyl cis-trans isomerase FKBP62	10
2.		Peptidyl-prolyl cis-trans isomerase CYP40	7
3.		Peptidyl-prolyl cis-trans isomerase PASTICCINO1	4
4.		Peptidyl-prolyl cis-trans isomerase FKBP42	2
5.		Peptidyl-prolyl cis-trans isomerase FKBP65	2
6.		70 kDa peptidyl-prolyl isomerase	2
7.	Hsp70-Hsp90 organising protein	Hsp70-Hsp90 organizing protein 1	2
8.		Hsp70-Hsp90 organizing protein 2	5
9.		Hsp70-Hsp90 organizing protein 3	17
10.	Anaphase-promoting complex subunit	Anaphase-promoting complex subunit 6	1
11.		Anaphase-promoting complex subunit 7	5
12.		Anaphase-promoting complex subunit 8	2
13.	UDP-N-acetylglucosamine--peptide N-acetylglucosaminyltransferase	UDP-N-acetylglucosamine--peptide N-acetylglucosaminyltransferase SEC	17
14.		UDP-N-acetylglucosamine--peptide N-acetylglucosaminyltransferase SPINDLY	8
15.	Envelope/membrane related TPR repeat containing-protein	Outer envelope protein 61	9
16.		Outer envelope protein 64	3
17.		Alpha-soluble NSF attachment protein	11
18.		Translocon at the outer membrane of chloroplasts 64	2
19.	Other TPR repeat-containing protein	Peroxisome biogenesis protein 5	10
20.		Protein SGT1 homolog	10
21.		Ethylene-overproduction protein 1	8
22.		Protein SULFUR DEFICIENCY-INDUCED 1 {ECO:0000303|PubMed:19154231	8
23.		Clustered mitochondria protein	6
24.		Protein CTR9 homolog	7
25.		Protein TSS	8
26.		Suppressor of RPS4-RLD 1	8
27.		Protein SLOW GREEN 1	6
28.		FAM10 family protein At4g22670	5
29.		Cell division cycle protein 27 homolog B	5
30.		Protein high chlorophyll fluorescent 107 {ECO:0000312|EMBL:AEE75897.1}	4
31.		Mitochondrial fission 1 protein A	4
32.		ALBINO3-like protein 3	3
33.		Coatomer subunit epsilon-1	3
34.		DnaJ protein P58IPK homolog	3
35.		ERAD-associated E3 ubiquitin-protein ligase component HRD3	3
36.		Serine/threonine-protein phosphatase 5	3
37.		Inactive TPR repeat-containing thioredoxin TTL3	3
38.		Coatomer subunit alpha-2	2
39.		F-box protein At1g70590	2
40.		Protein FLUORESCENT IN BLUE LIGHT	2
41.		Serine/threonine-protein kinase TOR	2
42.		TPR repeat-containing thioredoxin TTL4	2
43.		Probable serine/threonine-protein kinase At5g41260	2
44.		E3 ubiquitin-protein ligase CHIP {ECO:0000305}	1
45.		Protein LOW PSII ACCUMULATION 1	1
46.		Protein POLLENLESS 3-LIKE 2	1
47.		Equilibrative nucleotide transporter 8	1
48.	Unclear/unknown	Uncharacterised protein ycf37	2

49.		Uncharacterised TPR repeat-containing protein	3
50.		No match	51
		Total	288

Table S7: EF-Hand repeat-containing proteins identified in the calyx transcriptome of roselle.

No.	Protein group	Protein	Number of transcripts
1.	Calcium-dependent protein kinase	Calcium-dependent protein kinase 1	1
2.		Calcium-dependent protein kinase 10	1
3..		Calcium-dependent protein kinase 13	5
4		Calcium-dependent protein kinase 16	3
5.		Calcium-dependent protein kinase 2	3
6.		Calcium-dependent protein kinase 21	1
7.		Calcium-dependent protein kinase 28	3
8.		Calcium-dependent protein kinase 29	3
9.		Calcium-dependent protein kinase 3	7
10.		Calcium-dependent protein kinase 30	3
11.		Calcium-dependent protein kinase 32	2
12.		Calcium-dependent protein kinase 33	1
13.		Calcium-dependent protein kinase 4	4
14.		Calcium-dependent protein kinase 6	1
15.		Calcium-dependent protein kinase 7	4
16.		Calcium-dependent protein kinase 9	2
17.		Calcium-dependent protein kinase SK5	2
18.	Calcium-binding protein	Calcium-binding protein CML10	2
19.		calcium-binding protein CML13	2
20.		Calcium-binding protein CML19	1
21.		Calcium-binding protein CML2	1
22.		calcium-binding protein CML21	1
23.		calcium-binding protein CML22	1
24.		calcium-binding protein CML23	1
25.		Calcium-binding protein CML27	3
26.		Calcium-binding protein CML35	3
27.		Calcium-binding protein CML41	2
28.		Calcium-binding protein CML42	1
29.		Calcium-binding protein CML45	3
30.		Calcium-binding protein CML48	1
31.		Calcium-binding protein CML49	5
32.		Calcium-binding protein PBP1	2
33.	Calcineurin B-like protein	Calcineurin B-like protein 1	2
34.		Calcineurin B-like protein 10	9
35.		Calcineurin B-like protein 2	1
36.		Calcineurin B-like protein 3	12
37.		Calcineurin B-like protein 4	4
38.	Calmodulin	Calmodulin	3
39.		Calmodulin-2/4	2
40.		Calmodulin-5	1
41.	Calmodulin-like protein	Calmodulin-like protein 1	2
42.		Calmodulin-like protein 11	1
43.		Calmodulin-like protein 3	4
44.		Calmodulin-like protein 4	4
45.		Calmodulin-like protein 5	1
46.		Calmodulin-like protein 7	2
47.		Calmodulin-like protein 8	2
48.		Calmodulin-related protein	4
49.	Other EF-hand-containing protein	Calcineurin subunit B	4
50.		Caltractin	5
51.		Calumenin	4

52.		Serine/threonine protein phosphatase 2A regulatory subunit B''alpha	1
53.		Serine/threonine protein phosphatase 2A regulatory subunit B''beta	2
54.		Serine/threonine protein phosphatase 2A regulatory subunit B''delta	3
55.		Serine/threonine protein phosphatase 2A regulatory subunit B''gamma	1
56.		serine/threonine-protein phosphatase 2A regulatory subunit B	2
57.		External alternative NAD(P)H-ubiquinone oxidoreductase B1	2
58.		External alternative NAD(P)H-ubiquinone oxidoreductase B2, mitochondrial	3
59.		Two pore potassium channel c	3
60.		Two-pore potassium channel 5	3
61.		Mitochondrial Rho GTPase 1	4
62.		Mitochondrial Rho GTPase 2	9
63.		Respiratory burst oxidase homolog protein A	4
64.		Respiratory burst oxidase homolog protein C	1
65.		EH domain-containing protein 1	1
66.		EH domain-containing protein 2	3
67.		GTP diphosphokinase CRSH	1
68.		Lysophospholipid acyltransferase LPEAT2	3
69.		Phosphatidylserine decarboxylase proenzyme 2	1
70.		Phosphatidylserine decarboxylase proenzyme	1
71.	Unclear/unknown	No match	25
72.		Uncharacterised protein	10
		Total	225

Table S8: Armadillo repeat-containing proteins identified in the calyx transcriptome of roselle.

No.	Protein group	Protein	Number of transcripts
1.	U-box domain-containing protein	U-box domain-containing protein 2	1
2.		U-box domain-containing protein 3	12
3.		U-box domain-containing protein 4	21
4.		U-box domain-containing protein 4	2
5.		U-box domain-containing protein 9	1
6.		U-box domain-containing protein 11	2
7.		U-box domain-containing protein 12	2
8.		U-box domain-containing protein 13	7
9.		U-box domain-containing protein 14	2
10.		U-box domain-containing protein 15	1
11.		U-box domain-containing protein 17	2
12.		U-box domain-containing protein 18	1
13.		U-box domain-containing protein 19	1
14.	Armadillo repeat-containing kinesin-like protein	Armadillo repeat-containing kinesin-like protein 1	8
15.		Armadillo repeat-containing kinesin-like protein 2	23
16.		Armadillo repeat-containing kinesin-like protein 3	10
17.	Importin subunit	Importin subunit alpha-1	7
18.		Importin subunit alpha-1a	11
19.		Importin subunit alpha-1b	8
20.		Importin subunit alpha-2	7
21.		Importin subunit alpha-4	5
22.		Importin subunit alpha-9	1
23.	Other ARM repeat-containing protein	Protein ARABIDILLO 1	14
24.		Protein ARABIDILLO 2	2
25.		ARM REPEAT PROTEIN INTERACTING WITH ABF2	13
26.		Armadillo repeat-containing protein LFR	10
27.		Phospholipase A I	4
28.		Vacuolar protein 8	3
29.	Unclear/unknown	No match	14
		Total	195

Table S9: HEAT repeat-containing proteins identified in the calyx transcriptome of roselle.

No.	Protein	Number of transcripts
1.	Protein SHOOT GRAVITROPISM 6	52
2.	Serine/threonine-protein phosphatase 2A 65 kDa regulatory subunit A beta isoform	8
3.	26S proteasome non-ATPase regulatory subunit 1 homolog A	4
4.	Phosphoinositide 3-kinase regulatory subunit 4	1
5.	Transcription initiation factor TFIID subunit 6	1
6.	Microtubule-associated protein TORTIFOLIA1	1
7.	Microtubule-associated protein SPIRAL2-like	1
8.	CLIP-associated protein	8
9.	Coatomer subunit gamma-2	8
10.	Importin subunit beta-1	7
11.	Protein MOR1	7
12.	Protein VAC14 homolog	5
13.	Deoxyhypusine hydroxylase	5
14.	Beta-adaptin-like protein C	3
15.	AP-1 complex subunit gamma-2	1
16.	Importin subunit beta-3	1
17.	Putative uncharacterised protein	1
	Total	114
